# Impact of AHR Ligand TCDD on Human Embryonic Stem Cells and Early Differentiation

**DOI:** 10.3390/ijms21239052

**Published:** 2020-11-28

**Authors:** Indrek Teino, Antti Matvere, Martin Pook, Inge Varik, Laura Pajusaar, Keyt Uudeküll, Helen Vaher, Annika Trei, Arnold Kristjuhan, Tõnis Org, Toivo Maimets

**Affiliations:** 1 Chair of Cell Biology, Institute of Molecular and Cell Biology, University of Tartu, Riia 23, 51010 Tartu, Estonia; antti.matvere@ut.ee (A.M.); martin.pook@ut.ee (M.P.); inge.varik@gmail.com (I.V.); laura.pajusaar@gmail.com (L.P.); keyt.uudekyll@gmail.com (K.U.); helen.vaher@ut.ee (H.V.); annika.trei@ut.ee (A.T.); arnold.kristjuhan@ut.ee (A.K.); toivo.maimets@ut.ee (T.M.); 2 Chair of Biotechnology, Institute of Molecular and Cell Biology, University of Tartu, Riia 23, 51010 Tartu, Estonia; tonis.org@ut.ee; 3 Institute of Genomics, University of Tartu, Riia 23b, 51010 Tartu, Estonia

**Keywords:** aryl hydrocarbon receptor, human embryonic stem cells, neural progenitors, early mesoderm, definitive endoderm, TCDD, RNA-seq, ChIP-seq, ATAC-seq

## Abstract

Aryl hydrocarbon receptor (AHR) is a ligand-activated transcription factor, which mediates the effects of a variety of environmental stimuli in multiple tissues. Recent advances in AHR biology have underlined its importance in cells with high developmental potency, including pluripotent stem cells. Nonetheless, there is little data on AHR expression and its role during the initial stages of stem cell differentiation. The purpose of this study was to investigate the temporal pattern of AHR expression during directed differentiation of human embryonic stem cells (hESC) into neural progenitor, early mesoderm and definitive endoderm cells. Additionally, we investigated the effect of the AHR agonist 2,3,7,8-tetrachlorodibenzo-*p*-dioxin (TCDD) on the gene expression profile in hESCs and differentiated cells by RNA-seq, accompanied by identification of AHR binding sites by ChIP-seq and epigenetic landscape analysis by ATAC-seq. We showed that AHR is differentially regulated in distinct lineages. We provided evidence that TCDD alters gene expression patterns in hESCs and during early differentiation. Additionally, we identified novel potential AHR target genes, which expand our understanding on the role of this protein in different cell types.

## 1. Introduction

Aryl hydrocarbon receptor (AHR) is a ligand-dependent transcription factor belonging to the bHLH/PAS family of proteins. The unliganded AHR resides in the cytoplasm in complex with HSP90, AIP and p23. Upon activation by an agonist, AHR translocates to the nucleus where it is released from its cytoplasmic chaperones and dimerises with its nuclear partner ARNT (AHR nuclear translocator) [1,2]. This heterodimeric complex recognises and binds response elements in regulatory regions of its target genes, recruits cofactors and thereby modulates gene expression. Initially, AHR was identified as the mediator of toxic effects of various environmental contaminants, like polycyclic and halogenated aromatic hydrocarbons, including the most potent agonist, 2,3,7,8-tetrachlorodibenzo-p-dioxin (TCDD) [3]. Activation of AHR by these chemicals leads to induction of various xenobiotic metabolising enzymes such as CYP1A1 and CYP1B1, which are responsible for the degradation and elimination of these compounds from the cells. Later studies, however, have established that AHR can be activated by various endogenous and natural ligands, ascribing it an important role in cellular homeostasis, including regulation of the cell cycle, apoptosis, etc. The significance of AHR has been underscored, among others, in reproduction, liver homeostasis and the immune system [4,5,6]. In addition, recent advances in the field have emphasised the role of AHR in cells with high developmental potential i.e., stem cells.

Several studies have investigated the role of AHR in adult stem cells using known antagonists or agonists such as TCDD. Hematopoietic stem cells (HSC), which descend from the mesoderm, have high therapeutic value but are limited in expansion capacities in vitro. Advances in this field, however, have shown that the AHR antagonist StemRegenin1 promotes expansion of HSC, retaining their developmental potential [7]. In addition, the RNA-binding protein Musashi-2 (MSI2) was shown to induce HSC self-renewal [8]. It was later established that MSI-2 exerts its effect through silencing of AHR signalling, with reduction of *CYP1B1* expression being the key mechanism [9]. In addition, differentiation of mouse embryonic stem cells into cardiomyocytes has been shown to be interfered with by TCDD [10,11]. Accordingly, it was recently established that TCDD also disrupts differentiation of human embryonic stem cells (hESCs) into cardiomyocytes [12]. Interestingly, it was shown that the most profound effects appeared when TCDD-treatment was performed on hESCs rather than later during differentiation.

Studies investigating the role of AHR in neural differentiation have revealed that activation of Ahr by TCDD suppresses neural precursor cell proliferation in a mouse model [13]. The mechanism included impaired G1/S cell cycle transition, downregulation of cyclin D1 and upregulation of p27 (CDKN1B) protein levels. The impact of TCDD on neuronal differentiation has been further elucidated during differentiation of human embryonic stem cells. It was shown that temporal exposure to TCDD increases neural rosette formation and the number of cells positive for MAP2 and TH, the latter being a key enzyme in dopamine synthesis. Additional studies have investigated AHR during endodermal differentiation. TCDD was found to impair hESC differentiation into the pancreatic lineage and alter DNA methylation [14]. Importantly, hypermethylation of *PRKAG1*, a regulator of insulin secretion, was observed.

Embryonic stem cells (ESCs) are derived from the inner cell mass of a blastocyst. They are pluripotent, meaning that they can differentiate into three primordial lineages—ectoderm, endoderm and mesoderm—and give rise to every cell type of the body. Pluripotency is governed by specific transcription factors centred on OCT4, SOX2 and NANOG that enhance their own expression and suppress genes essential for differentiation. During differentiation of ESCs, pluripotency genes are generally downregulated, enabling upregulation of lineage-specific genes and thus lineage-specification. Differentiation of ESCs can be induced chemically, using small molecule compounds, as well as with growth factors, being also used in various combinations in commercially available differentiation media. In addition, formation of embryoid bodies (EBs), 3D structures that mimic in vivo differentiation, has been applied. Previous publications investigating AHR during the first steps of development have established that mouse Ahr is expressed at the 1-cell stage, downregulated at 2- and 8-cell stages followed by upregulation at later stages [15,16]. In mouse embryonic stem cells (mESCs), *Ahr* is repressed and upregulated during differentiation into EBs [17]. Importantly, Oct4, Sox2 and Nanog were shown to bind the distal promoter of *Ahr*, suppressing its expression and thereby promoting mESC mitotic progression and maintenance of pluripotency [18]. In human, *AHR* is expressed from the 1-cell stage throughout the blastocyst stage. *AHR* expression has been detected in various human embryonic stem cell (hESC) lines [19]. Importantly, AHR was reported to maintain the pluripotent state of hESCs and induce proliferation via kynurenine, an endogenous ligand of AHR, generated from tryptophan by IDO1 and TDO2 [19,20]. Consistent with this, AHR expression was downregulated following induction of ectodermal differentiation, ascribing AHR an important role in pluripotent hESCs as well as during differentiation [19].

There is little data about the temporal pattern of AHR during hESC differentiation. Considering this, our aims were to characterise AHR expression during induced differentiation of hESCs into neural progenitor, definitive endoderm and early mesoderm cells. Additionally, we evaluated the impact of TCDD on the transcriptome of hESCs as well as early differentiation events by RNA-seq, coupled with identification of genome-wide AHR binding sites by ChIP-seq and analysis of chromatin accessibility by ATAC-seq. We expand the knowledge on the role of AHR in stem cells by identifying its novel potential target genes by ChIP-seq coupled with changes in gene expression determined by RNA-seq.

## 2. Results

### 2.1. AHR Is Downregulated during Non-Directed Differentiation into Embryoid Bodies (EBs)

**Figure 1:**
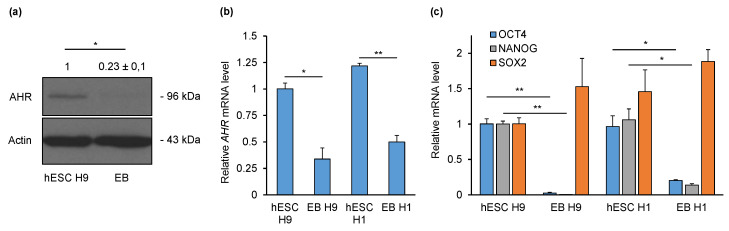
Expression of AHR and pluripotency marker genes in hESCs and embryoid bodies (EBs) cultured for 5 days. (**a**) Representative Western blot and densitometry analysis of AHR and actin protein levels in H9 cells. qPCR analysis of *AHR* (**b**), *OCT4*, *SOX2* and *NANOG* (**c**) mRNA levels in H1 and H9 hESCs and EBs. Data are presented relative to hESC H9 as means ± SEM from three independent experiments. * *p* < 0.05; ** *p* < 0.01.

To gain first insight into the regulation of AHR during early differentiation, we initially performed non-directed differentiation of H9 hESCs into EBs. Western blot analysis showed that AHR is expressed in hESCs and downregulated in EBs differentiated for five days (Figure 1a). Densitometry analysis revealed a four-fold reduction in AHR protein levels. Analysis of *AHR* mRNA expression by qPCR revealed a three-fold downregulation in EBs compared to hESCs (Figure 1b). A similar expression pattern was also detected with another hESC line—H1 and its EBs. Differentiation of hESCs was validated by expression analysis of core pluripotency markers—*OCT4*, *SOX2* and *NANOG*. As expected, *OCT4* and *NANOG* mRNAs were downregulated in both H9 and H1 EBs compared to their respective pluripotent hESCs (Figure 1c). The levels of *SOX2*, however, remained unchanged, indicating neural differentiation as a prominent lineage [21]. Collectively, we showed that AHR is downregulated during non-directed differentiation in both H9 and H1 cell lines. This indicates a broader biological, rather than single cell line-specific significance to the downregulation of AHR in the early steps of pluripotent cell differentiation.

### 2.2. AHR Expression Shows Distinct Patterns during Directed Differentiation into Three Lineages

**Figure 2:**
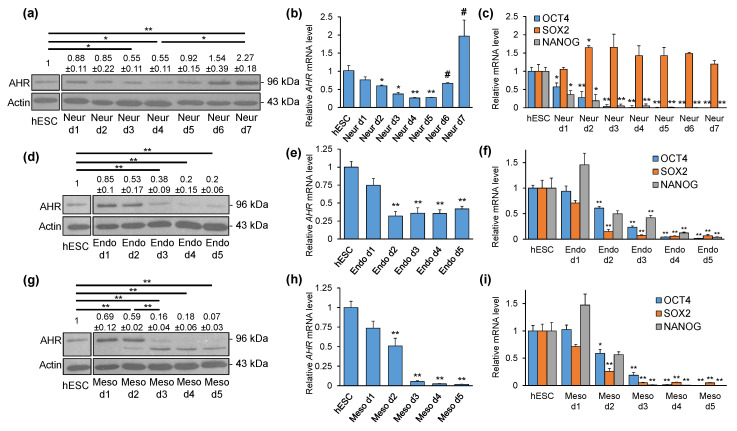
Analysis of AHR and pluripotency marker gene expression in H9 hESCs and during directed differentiation into neural, endo- and mesodermal lineages. (**a**) Representative Western blot and densitometry analysis of AHR and actin protein levels during differentiation into neural lineage. qPCR analysis of *AHR* (**b**), *OCT4*, *SOX2* and *NANOG* (**c**) mRNA levels. (**d**) Representative Western blot and densitometry analysis of AHR and actin protein levels during differentiation into endodermal lineage. qPCR analysis of *AHR* (**e**), *OCT4*, *SOX2* and *NANOG* (**f**) mRNA levels. (**g**) Representative Western blot and densitometry analysis of AHR and actin protein levels during differentiation into mesodermal lineage. qPCR analysis of *AHR* (**h**), *OCT4*, *SOX2* and *NANOG* (**i**) mRNA levels. Data are presented relative to hESCs as means ± SEM from three independent experiments. * *p* < 0.05; ** *p* < 0.01 vs. hESCs; # *p* < 0.05 vs. Neur d5.

We next aimed to characterise the temporal pattern of AHR expression during hESC differentiation into three lineages. For this, H9 hESCs were differentiated into neural progenitor, definitive endoderm and early mesoderm cells using commercial differentiation kits. Western blot and densitometry analyses showed a 45% reduction in AHR protein levels after three (d3) and four days (d4) neural differentiation when compared to undifferentiated hESCs (Figure 2a). Compared to d4, AHR levels showed an increasing trend at d5 and d6 reaching a statistically significant increase at d7. Interestingly, d7 neural cells exhibited 2.27-fold elevation of AHR protein compared to hESCs. Analysis of *AHR* mRNA expression revealed a somewhat similar pattern. *AHR* downregulation occurred at d2 of differentiation and persisted throughout d5 (Figure 2b). Compared to d5, *AHR* expression increased significantly at d6 and d7, reaching a 2-fold, although statistically insignificant, upregulation relative to hESCs. Differentiation of hESCs was validated by gene expression analysis of core pluripotency and lineage-specific markers. As expected, *OCT4* and *NANOG* expression started to decline from d1 of differentiation (Figure 2c). Additionally, the persisted expression of *SOX2* indicated neural differentiation. Analysis of *PAX6* and *OTX2* expression corroborated neural differentiation, as their expression started to increase at d1 and d2 of differentiation, respectively, and remained relatively high throughout the experiments (Figure S1a,b).

Differentiation of hESCs into definitive endoderm cells revealed that AHR protein is reduced 62% by d3 and 80% by d4 and d5 (Figure 2d). *AHR* mRNA levels started to decrease from d2 (−68%) and remained at similar levels throughout d5 (d3 −64%, d4 −65%, d5 −58%) (Figure 2e). Expression of each of the pluripotency genes *OCT4*, *SOX2* and *NANOG* was downregulated by d2 and continued to decrease until d5 (Figure 2f). Endodermal differentiation was confirmed by the lineage-specific marker genes *SOX17* and *GATA4*, which were detected at d2 of differentiation and increased at later time points (Figure S1c,d).

Mesodermal differentiation showed a 41% reduction of AHR protein levels at d2 followed by more robust downregulation by d3 (−84%), d4 (−82%) and barely detectable AHR at d5 (−93%) (Figure 2g). Similar dynamic changes were observed in *AHR* mRNA expression. There was a 49% reduction of *AHR* mRNA by d2 followed by a more robust silencing at d3–d5 (d3 −95%, d4 −93%, d5 −98%) (Figure 2h). Confirming differentiation, *OCT4, SOX2* and *NANOG* were downregulated starting from d2 (Figure 2i). Accordingly, the mesoderm-specific markers *T* and *HAND1* were upregulated starting from d2 and remained relatively high until d5 (Figure S1e,f). Taken together, these results illustrate that AHR is significantly downregulated during early differentiation with distinct patterns and extents of reduction in different lineages.

### 2.3. TCDD Does Not Affect Pluripotency- or Differentiation-Specific Marker Gene Expression

We next sought to determine whether the environmental contaminant TCDD might affect the pluripotency of hESCs. In non-treated, DMSO or TCDD (10 nM) treated cells (3 days), the proportion of OCT4^+^SOX2^+^NANOG^+^ cells was evaluated by flow cytometry. We observed no effect of DMSO or TCDD on hESC pluripotency when compared to non-treated cells (Figure S2a). Accordingly, the mRNA levels of *OCT4*, *SOX2* and *NANOG* remained unaffected (Figure S2b). In addition, TCDD significantly upregulated *CYP1A1*, the classical AHR target gene, thereby confirming the functionality of the AHR pathway in these cells (Figure S2c).

**Figure 3:**
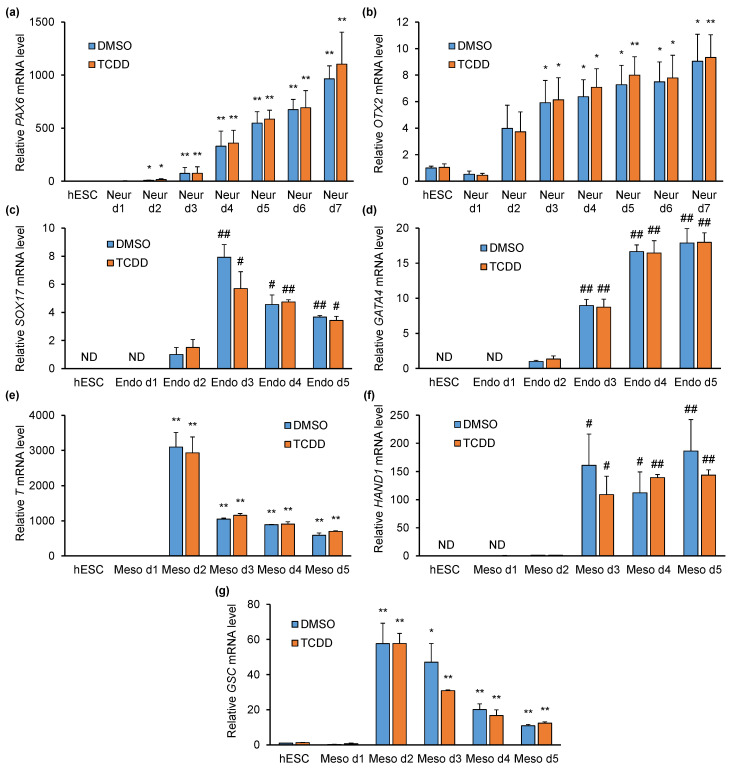
Analysis of lineage-specific marker gene expression in DMSO or TCDD pre-treated H9 hESCs and during directed differentiation into neural, endo- and mesodermal lineages. qPCR analysis of neural marker genes *PAX6* (**a**) and *OTX2* (**b**), endodermal marker genes *SOX17* (**c**) and *GATA4 (***d***)* and mesodermal marker genes *T (***e***)*, *HAND1 (***f***)* and *GSC (***g***)* mRNA levels. Data are presented relative to hESC DMSO (**a**,**b**,**e**,**g**) or d2 DMSO (**c**,**d**,**f**) as means ± SEM from three independent experiments. * *p* < 0.05 vs. hESC; ** *p* < 0.01 vs. hESC; # *p* < 0.05 vs. d2; ## *p* < 0.01 vs. d2.

Our next aim was to investigate whether TCDD affects early differentiation of hESCs. Previous publications have emphasised the effect of TCDD pre-treatment (i.e., prior to differentiation at the pluripotent stage) [12,14]. Thus, hESCs were pre-treated with control (DMSO) or TCDD (10 nM) for three days followed by differentiation with commercial media in the presence of DMSO or TCDD (10 nM). Neural differentiation in the presence of DMSO similarly resulted in the upregulation of *PAX6* and *OTX2*, as presented above (Figure 3a,b; Figure S1a,b). TCDD had no effect on these genes, as the expression levels were comparable to DMSO-treated cells. Additionally, we observed no effect of TCDD on *AHR* or *OCT4*, *SOX2* and *NANOG* expression (Figure S3a–d). AHR target gene *CYP1A1*, however, was upregulated in the presence of TCDD (Figure S3e). Endodermal differentiation in the presence of DMSO resulted in upregulation of the lineage-specific markers *SOX17* and *GATA4* starting from day 2 (Figure 3c,d). Again, TCDD had no significant effect on the expression of these genes, as the levels were comparable to DMSO-treated cells. In addition, the levels of *AHR*, *OCT4*, *SOX2* and *NANOG* were not influenced by TCDD (Figure S4a–d). Mesodermal differentiation in the presence of DMSO resulted in upregulation of the specific marker genes *T*, *HAND1* and *GSC* starting from day 2, and TCDD did not influence their expression (Figure 3e–g). As with other lineages, the expression of *AHR*, *OCT4*, *SOX2* and *NANOG* were not influenced by TCDD treatment (Figure S5a–d). Collectively, these results indicate that TCDD does not interfere with the expression of classical lineage marker genes. However, this does not necessarily eliminate the possibility that TCDD has adverse effects on hESC differentiation and thus needs scrutiny.

### 2.4. Impact of TCDD on hESCs and Early Differentiation

**Figure 4:**
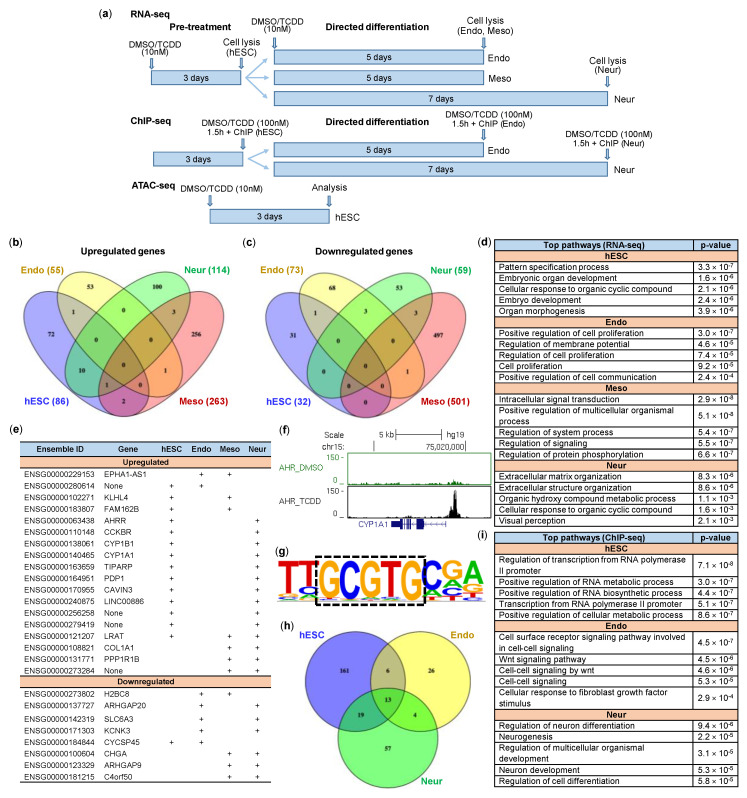
Genome-wide analysis of possible AHR target genes in H9 hESCs and differentiated cells. (**a**) Experimental design. RNA-seq analysis of upregulated (**b**) and downregulated (**c**) genes in TCDD vs. DMSO pre-treated H9 hESCs and differentiated cells (fold change > 1.2, *p*-value < 0.05). (**d**) Gene ontology analysis of all differentially regulated genes showing top five biological pathways influenced by TCDD. (**e**) Differentially regulated (“+”, fold change > 1.2, *p*-value < 0.05) genes overlapping between hESCs and differentiated cells. (**f**) ChIP-seq analysis was performed to find AHR-bound regions. AHR binding profile near classical AHR target gene *CYP1A1.* Data is presented as fragment pileup and visualized in the UCSC Genome browser. (**g**) HOMER motif discovery software was used for motif analysis in AHR-bound regions. (**h**) Venn diagram showing number of AHR-bound regions in hESCs and differentiated cells. (**i**) Gene ontology analysis of genes associated with lineage-specific AHR-bound regions showing top five biological pathways.

In order to gain a more global understanding of the impact of TCDD on early differentiation, we next performed high-throughput mRNA-sequencing (RNA-seq). Gene expression in hESCs was analysed after 3 days of DMSO/TCDD (10nM) treatment. Additionally, the pre-treated hESCs were induced to differentiate into neural progenitor (7 days), definitive endoderm (5 days) and early mesoderm (5 days) cells in the presence of DMSO or TCDD (Figure 4a). Our results show that TCDD altered gene expression profiles in hESCs and differentiated cells, with most prominent changes in mesodermal lineage (Figure S6). More precisely, TCDD upregulated 86 genes (*p* < 0.05) in hESCs (Figure 4b). Fifty-five genes were upregulated in endodermal, 263 genes in mesodermal and 114 genes in neural cells (Figure 4b). TCDD treatment resulted in downregulation of 32 genes in hESCs. In addition, 73 genes were downregulated in definitive endoderm cells, 501 genes in mesodermal cells and 59 genes in neural progenitors (Figure 4c). The complete list of differentially expressed genes can be found in Supplementary File S1. Importantly, RNA-seq corroborated our results obtained from RT-qPCR experiments described above. Gene ontology analysis with all differentially expressed genes (up- and downregulated) was performed to find top biological pathways influenced by TCDD (Figure 4d). In hESCs, the effect of TCDD was most apparent in regulating genes associated with pattern specification, embryonic organ development and organ morphogenesis, signifying the relevance of AHR in these key processes in early development. In endoderm cells, TCDD influenced genes associated with positive regulation of cell proliferation and regulation of membrane potential. During mesodermal differentiation, TCDD affected genes associated with intracellular signal transduction, whereas TCDD influenced extracellular matrix and structure organisation in neural differentiation. Some TCDD-influenced pathways were common in different cell types, e.g., cellular response to organic cyclic compounds in hESCs and neural cells. However, the overall results revealed distinct patterns between lineages, implying AHR to have time/cell/lineage-specific roles during early differentiation. This assumption was supported by our findings that among differentially regulated genes by TCDD, only a small proportion was common to multiple cell types (Figure 4e). These included well-recognized AHR targets such as *CYP1A1*, *CYP1B1*, *TIPARP* and *AHRR*, but also some less-characterized genes or novel non-coding RNA genes (Figure 4e).

We next sought to determine which genes might be direct targets of AHR. For this, we performed ChIP-seq experiments with hESCs, neural progenitor and definitive endoderm cells following 1.5 h exposure to TCDD (100 nM) or DMSO. Early mesoderm cells were excluded from these experiments, since AHR protein was essentially absent in these cells (Figure 2g). ChIP-qPCR with known AHR target genes was carried out for quality control before and after library preparation (Figures S7–S9). AHR binding nearby its classical target genes *CYP1A1* (Figure 4f), *AHRR*, *TIPARP* and *CYP1B1* (Figure S10), visualised in the UCSC genome browser, was shown as proof of the quality of the ChIP-seq experiment. DNA motif analysis of combined enriched regions in ChIP-seq of 3 cell-types uncovered AHR core sequence 5’-GCGTG-3’ as the top enriched motif being present in 57% of target sequences (Figure 4g). Additional transcription factor motifs associated with enriched regions can be found in Figure S11. We identified 199 AHR-bound regions in hESCs, 93 in neural progenitor and 49 in definitive endoderm cells (Figure 4h). Genes associated (within +/−200 kb of TSS) with overlapping binding sites are listed in Table S1. Primary biological pathways linked to AHR-bound genes in hESCs are regulation of transcription and positive regulation of RNA metabolic and biosynthesis processes (Figure 4i). Top pathways after early endodermal differentiation appear to be involved in cell–cell and WNT signalling, whereas AHR-bound genes in neural progenitor cells are mostly related to neurogenesis (Figure 4i).

Our next aim was to gain insight into possible direct AHR target genes in embryonic stem cells. For this, we sorted out differentially expressed genes that were associated with AHR-binding (Supplementary File S2). We found 28 upregulated genes, which also had TCDD-induced AHR binding within +/−200kbp of TSS (Table 1), suggesting that AHR may be involved in direct transcriptional activation of these genes. In addition to known AHR targets, the list contains several genes, which are recognised for their roles in embryonic development, e.g., *SIX3* and *SIX6* involved in regulation of multipotent neuroretinal progenitors [22], *LRAT* in regulating retinoid homeostasis during embryogenesis [23], *RORA* in regulating the differentiation and survival of Purkinje cells [24] and *LHX4* in the control of differentiation and development of the pituitary gland [25]. However, their interactions with AHR have not been described previously.

When combining RNA-seq and ChIP-seq data in endodermal cells, there was no overlap between genes associated with AHR binding and genes with differential expression. Four of the differentially expressed genes in endoderm cells were AHR-bound in hESCs. TCDD positively regulated the expression of *CDCA7L* and *S1PR1*, whereas substantial downregulation of *MYADML2* and *CUZD1* was observed (Table 2). Remarkably, the latter were upregulated in hESCs, again reinforcing the previously ascribed role of the AHR as being a protein with tissue-specific functions. Mesodermal differentiation showed distinct expression level for 16 genes between DMSO vs. TCDD treated cells, associated with AHR binding in hESCs, Endo or Neur cells (Table 3). Interestingly, 10 of these, including *CYP1A1*, were downregulated upon TCDD treatment compared to cells treated with DMSO. Considering essentially non-detectable AHR protein level in these cells (Figure 2), this outcome may be the result of secondary effects elicited by TCDD-AHR signalling during the pluripotent stage.

Gene expression analysis of AHR-bound genes in neural progenitor cells expectedly showed upregulation of AHR target genes, with most noticeable change in the expression of *CYP1A1* (35-fold). While most of the genes (Table 4) had TCDD-induced AHR-binding in both hESC and neural progenitors (including *LRAT* and *LINC00886*), the expression of *GRB7*, *IKZF3*, *CYP27A1*, *CCDC60* and *TSC22D1* were specific to the latter. TCDD also upregulated *CCKBR* and *CDX2*, which in some ways illustrates the potential severity of dioxin toxicity, since these are recognised as trophectoderm markers [26].

As TCDD has been previously shown to influence gene expression by epigenetic changes, we next performed ATAC-seq (assay for transposase accessible chromatin followed by high-throughput sequencing) analysis [14]. ATAC-seq was performed with pre-treated hESCs (3 days DMSO or 10 nM TCDD), since this is the time window when cells are most susceptible to TCDD during early development [12,27]. Overall, we found 157 genomic regions that were differentially affected by TCDD in terms of chromatin condensation (Supplementary File S2). Our results revealed eight regions with reduced accessibility in response to TCDD compared to DMSO-treated cells. However, the expression of six genes (*CRYM*, *GFOD1*, *GLRX5*, *NPIPB3*, *SIRT5*, *TCL1B*) that were associated with these regions did not change. On the other hand, TCDD increased chromatin accessibility within 149 regions associated with 222 genes. Eleven of these genes were associated with AHR binding within TSS proximity, including, for example, *ITGA6*, *RCC2*, *ZNF532*, *DLX2*, *MALT1* and *PADI4*. In several cases, increased chromatin accessibility and AHR binding were also accompanied by increased expression of the associated gene, hinting that AHR may be directly involved in activation of these genes in hESCs. Indeed, *TIPARP*, a well-known AHR target, was among these genes. In addition to previously mentioned *LRAT*, which is important in eye development/retinol metabolism pathway, possible novel AHR targets in hESCs include exocyst complex component gene *EXOC2*, but also less-characterised *LINC00886* (Supplementary File S2).

Although this study did not particularly focus on investigating the endogenous roles of the AHR, ChIP-seq analysis revealed several regions in control-treated hESCs or differentiated cells that were bound by AHR. We found AHR protein within 1 kbp of TSS of numerous genes with the effect being common to hESCs, definitive endoderm and neural progenitor cells (e.g., *BAD*, *NUDT3*, *DAGLA*, *DICER1*, *CBX3*, *HNRNPA2B1*, *STRBP*, *SLC39A10*, *DDX17*, *TPRA1*, *SEPTIN7*, *EPC2*, *TIPARP*, *LINC00886*). A few of these, including *DICER1*, *HNRNPA2B1*, *NUDT3* and *DDX17*, are known to be involved in RNA processing (Figure S12). Indeed, regulation of RNA metabolic and biosynthesis processes were among the top biological pathways associated with AHR-bound genes, as shown by GO analysis (Figure 4h). Furthermore, AHR was found in the promoter of miR-302-367 gene cluster (Figure S13), which is known to regulate pluripotency [28,29]. In various cases, we found strong AHR binding only in hESCs (e.g., *NDUFAF7*, *NEUROG3*, *OPA1*, *BICD1*, *EIF4G2*, *FAM218A*, *HS3ST5*, *METTL9*, *CCDC34*, *ALDH1A1*, *PRICKLE1*, *ROR1*, *SMAD7*, *SPN*, *ETAA1*, *KCTD1*, *PRDM2*, *DUSP6*, *ACACA*, *ASAP1*, *RBPMS*, *SLC8B1*, *TPCN1*). The exact impact of AHR binding to these genes and possible endogenous roles of AHR in hESCs, however, need to be elucidated in future studies.

## 3. Discussion

The aryl hydrocarbon receptor was initially identified as the mediator of toxicity of a broad spectrum of environmental contaminants. In addition to its role in detoxification of these chemicals, AHR has also been shown to promote tumour formation and progression and teratogenesis. TCDD toxicity during embryonic development includes hydronephrosis and cleft palate. Importantly, these defects were absent in Ahr-deficient mice [30]. The incidence of cleft palate was later implicated with interference of TGFβ pathway, as exogenous TGFβ abolished this teratogenic effect of TCDD [31]. Epidemiologic studies have provided evidence that environmental contaminants like TCDD can cause congenital defects in newborns, indicating that TCDD interferes with early development [32]. Experiments in animals have shown that TCDD impairs cardiac differentiation, development and function, underscoring its impact on mesodermal differentiation [10,12,33]. Additional publications have implicated TCDD exposure in disruption of endodermal differentiation, and impairment of pancreatic homeostasis and type 2 diabetes [14,34,35]. In addition, TCDD has been associated with developmental neurotoxicity in both animal models as well as humans, including motor and cognitive deficits in TCDD-exposed rats [36,37,38]. Although numerous studies have investigated the result of TCDD-treatment in various cell types with reduced developmental potential, there is little data on its effect during the very first steps in human embryonic stem cell differentiation. Additionally, as relatively little attention has been paid on how AHR itself is regulated during early differentiation, we determined the temporal pattern of AHR during hESC differentiation into neural, endodermal and mesodermal lineages.

Previous publications on mouse embryonic stem cells have established that Ahr is essentially absent in the pluripotent stage followed by upregulation during non-directed differentiation into embryoid bodies. Our results reveal that in hESCs AHR is significantly expressed at both mRNA and protein levels. Contrarily to mESCs, AHR levels decrease during differentiation into embryoid bodies in both H9 and H1 human embryonic stem cell lines, implicating that this is characteristic of human cells rather than one cell line specific feature. Supporting this, a recent publication demonstrated that *AHR* is indeed expressed in a panel of hESC lines, whereas in EBs, *AHR* expression was decreased [19]. As embryoid bodies contain cells differentiating into all three lineages, it was our next objective to determine *AHR* expression during such differentiations. More precisely, we aimed to investigate the temporal pattern of AHR, rather than just look into the endpoints. To date, there are numerous protocols for hESC differentiation using small molecule compounds. However, we decided to use commercially available media that are thoroughly verified to result in highly reproducible and efficient differentiation. The use of such media were, according to the manufacturer’s instructions, described to result in differentiation of hESCs into neural progenitor, definitive endoderm and early mesoderm cells. Accordingly, in our experiments we analysed the expression of lineage specific markers, which were consistently upregulated, coupled with downregulation of pluripotency marker genes following differentiation. Analysis of *AHR* expression during neural differentiation revealed that it is downregulated from day 2 to day 5 of differentiation, followed by upregulation at day 6 and 7. Somewhat similar results were presented by Yamamoto and colleagues, who observed downregulation of *AHR* at the tested timepoints of day 3 and day 6 after ectoderm induction [19]. In addition, they postulated that AHR maintains the undifferentiated state of hESCs via its endogenous agonist kynurenine—the metabolite of tryptophan present in cell culture media of undifferentiated hESCs. Compared to neural cells, endodermal differentiation resulted in more profound downregulation of AHR, whereas mesodermal cells seemed to lack AHR. Interestingly, in our Western blot experiments with the AHR antibody, mesodermal differentiation resulted in a second band with lower molecular weight. There is a possibility that this may be the result of unspecific detection of an unknown protein by the AHR antibody. However, the inverse correlation with the 96kDa protein band suggests the possibility of a novel modification for AHR. Although we did not succeed in characterising this, the potential proteolytic cleavage of AHR and perseverance throughout mesodermal differentiation makes it an intriguing topic for further investigations. The downregulation of AHR during early differentiation into all three lineages seems to be in line with its proposed role in pluripotency maintenance. However, the differential regulation in distinct lineages ascribes AHR an important lineage-specific role.

As the TCDD-dependent derailment of differentiation in various cell types with high developmental potential has been characterised previously, one of our aims was to determine if TCDD might interfere with the early differentiation of hESCs. A recent study revealed that mesodermal differentiation is impaired by TCDD, as evidenced by decreased expression of the mesoderm marker gene *T* [12]. Notably, the effect of TCDD was most profound when TCDD-treatment was performed during the pluripotent stage of hESCs rather than during the first days of differentiation. Although the underlying cause was not investigated, it is reasonable to argue that this might be due to profound downregulation of AHR during mesodermal differentiation. Similarly, the pre-treatment was used in another study that investigated the effect of TCDD on endodermal differentiation [14]. Considering this, we treated hESCs for 3 days with TCDD or DMSO followed by differentiation into three lineages in the presence of TCDD or DMSO and evaluated their differentiation potential by analysing the expression of lineage-specific marker genes. Surprisingly, TCDD seemed to have no effect on early stage of hESC differentiation, as it did not significantly influence the expression of the selected marker genes *PAX6* and *OTX2* (neural), *SOX17* and *GATA4* (endodermal) and *T* and *HAND1* (mesodermal), in contrast to previous publications demonstrating TCDD-dependent downregulation of *SOX17* and *T* [12,14]. In addition, TCDD had no effect on the expression dynamics of OCT4, NANOG and SOX2. This, in turn, was unexpected as binding of AHR on the regulatory regions of these genes and modulation of expression has been demonstrated previously in both hESCs as well as cancer stem-like cells [19,39,40]. It is noteworthy, however, that these differences might be accountable on distinct cell lines, ligands and protocols used for differentiation.

To take a deeper look into the effects of TCDD, we next analysed its impact on global gene expression in hESCs, neural progenitors, definitive endoderm and early mesoderm cells. RNA-seq revealed that TCDD-treatment results in significant changes in gene expression, both up- and downregulation, in every cell type tested. Interestingly, the highest number of genes were dysregulated in the mesodermal lineage. Considering that AHR was almost absent in early mesoderm cells, this underscores the impact of TCDD in the pluripotent stage and/or during the very first days of differentiation. Recently, Fu and colleagues demonstrated that TCDD-treatment impairs mesodermal differentiation into cardiomyocytes [12]. Importantly, they observed downregulation of the key early mesoderm markers—*T* and *GSC*—and binding of AHR to their corresponding promoters two days after differentiation of pre-treated hESCs. Contrarily, our results show no impact of TCDD on these genes during the 5-day differentiation, as evidenced by RT-qPCR. Correspondingly, RNA-seq revealed no changes in their expression on day 5. Among the genes that they analysed (RT-qPCR) only two were affected by TCDD in our RNA-seq experiments—*HCN4* showed similar downregulation, whereas *TNNI3*, conversely, showed upregulation. Although these discrepancies remain unresolved, it is reasonable to argue that this may be the result of distinct differentiation protocols, as Fu and colleagues used CHIR99021 and B27 (minus insulin) supplement for mesodermal differentiation. However, the conclusion that TCDD has an effect on mesodermal differentiation, although via potentially different routes, is a common finding in both studies.

In hESCs, TCDD-treatment resulted in significant upregulation of LHX4 and SIX3, which are associated with cleft palate, the hallmark phenotype of TCDD toxicity [30,41,42]. Additionally, the promoters of *LHX4* and *SIX3* were bound by AHR following TCDD-treatment, indicating that AHR might directly upregulate these genes in hESCs. Comparison of differentially regulated genes revealed that hESCs and neural progenitor cells had the largest number of commonly upregulated genes, which is in line with relatively high *AHR* expression in these cells. Importantly, the classical AHR targets *TIPARP*, *AHRR*, *CYP1A1* and *CYP1B1* were commonly upregulated in hESCs and neural progenitors following TCDD-treatment. In meso- and endoderm, most of these genes remained unchanged, indicating that the profoundly low levels of AHR render it largely unresponsive to TCDD. This, in turn, hints that the disruption of differentiation in these lineages takes place in the pluripotent stage and/or during the first days of differentiation. Gene ontology analysis revealed that top pathways influenced by TCDD in hESCs are connected to organ and embryo development, being consistent with the disruptive effect of TCDD on the developing embryo [43].

DNA motif analysis revealed that only 57% of the AHR-bound regions contained the consensus AHR response element (AHRE) 5’-GCGTG-3’. This was expected, as previous publications have also described AHR-dependent regulation of genes lacking the AHRE [44,45]. Among others, AHR has been described to bind the tetranucleotide motif 5’-GGGA-3’, the E-box and binding sites for the transcription factors FOXA1 and SP1, emphasising the promiscuity of AHR in binding to DNA or possibly modulating gene expression via other proteins [46,47,48].

Gene ontology (GO) analysis with differentially expressed genes revealed that in hESCs TCDD influences biological processes associated with pattern specification, embryonic organ and embryo development. Among the influenced genes, several had nearby AHR binding—*PCDH8*, *LHX1*, *LRP4* and *SIX3*. Among these, *PCDH8*, *LHX1* and *LRP4* have been shown previously to be modulated by TCDD [49,50,51]. GO analysis with TCDD-treated endodermal cells revealed regulation of cell proliferation as the major influenced biological process. None of the genes dysregulated were associated with AHR binding in endodermal cells. However, *S1PR1* and *CDCA7L* had nearby AHR binding in hESCs, although their expression was not influenced at the pluripotent stage. Consistently, the impact of TCDD on *S1PR1* has been shown previously [52]. GO on mesodermal cells indicated that TCDD, among others, interferes with intracellular signal transduction and regulation of multicellular organismal processes. Analysis of genes associated with these biological processes revealed that *ADM*, *FGFR3*, *GADD45G* and *MAP3K8* had nearby AHR binding in hESCs. Consistently, regulation of *ADM* and *FGFR3* by TCDD has been demonstrated previously [53,54]. GO analysis performed on neural cells revealed extracellular matrix organisation and visual perception among top biological processes affected by TCDD. Among the underlying genes, *CYP1B1*, *CRYBB1* and *LRAT* had nearby AHR binding in neural cells or hESCs. The regulation of *CYP1B1* by AHR is well documented and is considered as a classical target, while only one study has shown regulation of *LRAT* by TCDD [55]. Collectively, these data illustrate that TCDD interferes with various biological processes lineage-dependently during the early development. The mechanism can include direct action of activated AHR on certain genes, but also via secondary mechanisms, i.e., through modulation of the expression of other genes.

Several health risks have been associated with TCDD exposure during foetal development, pointing to neurological effects elicited by TCDD. A recent study by Sarma et al. showed that TCDD increased the number of neuronal rosettes and TH-positive neuronal cells during the early stages of hESC differentiation, although the exact mechanisms remained elusive [27]. Our findings from genome-wide analyses may enlighten this matter, as we found several TCDD-regulated possible direct AHR target genes bound by AHR, which play a role in neural differentiation. *RORA* is highly expressed and developmentally regulated in many regions of the brain, including thalamus and Purkinje cells [24]. The critical role of *RORA* in neurogenesis is supported by its involvement in survival and differentiation of Purkinje cells and in regulating genes related to dendritic differentiation and the glutamatergic pathway [56]. In addition, TCDD-elicited effects on AHR binding, upregulation of mRNA expression and changes in chromatin condensation were common to *EXOC2* and *LRAT*. Importantly, AHR binding within regulatory regions of these genes was induced not only by TCDD, but was present in untreated cells, suggesting these may also be regulated endogenously by AHR in pluripotent cells. The importance of *EXOC2* (exocyst complex component) in human brain development has been demonstrated previously, as mutations in this gene cause severe developmental defects [57]. Specifically, pathogenic variants of *EXOC2* were associated with brain abnormalities including severe developmental delay, dysmorphism, poor motor skills and microcephaly. Interestingly, a recent study demonstrated that activation of AHR by its endogenous ligand kynurenine was a fundamental link in microcephaly caused by Zika virus [58].

Of likely novel AHR target genes in hESCs, one of the most highly upregulated was *LRAT*, which is a key gene involved in retinoid/visual cycle [23]. *LRAT* is highly expressed in the eye, and dysregulation of this gene has been associated with early onset retinal dystrophy [59]. In addition to gene ontology analysis, which showed visual perception among top biological pathways influenced by TCDD, oculomotor defects have been previously reported in *Ahr* knockout mice [60]. Moreover, AHR appeared to regulate *SIX6* and *SIX3*, genes that are expressed during development of the early stages of the visual system and that are required for the maintenance of multipotent retinal progenitor cells [22,61,62,63]. Additionally, Six3 was recently shown to be essential in determining cell-fate in neuroretinal cells [22].

Previously, activation of AHR by kynurenine has been demonstrated in hESCs, indicating that AHR is endogenously activated and linking AHR to maintenance of pluripotency [19]. Thus, the importance of AHR in embryonic stem cells is evident. In line with this, we observed common AHR binding on the regulatory regions of several genes in control-treated (DMSO) hESCs or differentiated cells, whereas differences in DNA binding with TCDD-treated cells were marginal. Interestingly, a common nominator between some of these genes seemed to be the relation to RNA processing events. As shown previously, regulation of RNA levels is a key mechanism regulating pluripotency [64]. Importantly, GO analysis with AHR-bound genes in hESCs revealed regulation of RNA metabolism and biosynthesis among top pathways. One of the underlying genes was miR-302, which is highly expressed in embryonic stem cells [65,66]. Interestingly, miR-302 has been shown to be inducible by AHR ligands during reprogramming of mouse somatic cells [67]. Thus, we hypothesise that one possibility how AHR might support pluripotency of hESCs is via positive regulation of miR-302. DICER1 is an important protein involved in bioprocessing of miRNAs and has also been shown to be essential for self-renewal of hESCs [64]. We observed strong binding of AHR on the promoter of *DICER1* in hESCs, further hinting on the importance of AHR in the maintenance of self-renewal, as suggested previously [19]. Importantly, AHR bound *DICER1* promoter also in differentiated cells, suggesting a new mechanism as to how AHR could regulate gene expression in different cells. Another potential target for AHR is *HNRNPA2B1*, as binding to its promoter was detected in every cell type treated with DMSO or TCDD. HNRNPA2B1 is an RNA-binding protein known for its role in primary miRNA processing and alternative splicing but has also been identified as a key player in miRNA sorting into exosomes [68,69]. Abundant AHR binding was also observed in the promoters of *NUDT3* and *DDX17*, which are involved in mRNA decapping and miRNA biogenesis [70,71]. Future studies with functional experiments (e.g., knocking out *AHR* or using known antagonists) should focus on investigating the exact function and underlying mechanisms of AHR in regulation of these genes in stem cells and during differentiation.

In addition to mediating the toxicity of environmental chemicals and regulating cellular homeostasis, AHR has been thoroughly investigated in cancer. In different cancer types, AHR expression and activity negatively correlates with patient survival, including glioblastoma and hepatocellular carcinoma [20,72]. In these cancers, several biomarkers have been described as predicting therapeutic outcomes, e.g., CDCA7L, BICD1, DDX17, S1PR1 and SEPT7. Importantly, we provide evidence that AHR might directly regulate these genes, thereby expanding our knowledge on the role of AHR in cancer. Collectively, we are the first to provide evidence on distinct lineage-specific regulation of AHR expression during human embryonic stem cell differentiation. In addition, we characterised the impact of the environmental contaminant TCDD on early differentiation and determined novel potential AHR target genes in pluripotent as well as differentiating cells. Thus, the presented data herein open interesting avenues in discovering novel roles of AHR in cellular homeostasis as well as disease.

## 4. Materials and Methods

### 4.1. Cell Culture

Human ES cell lines H1 (46, XY, WA01) and H9 (46, XX, WA09) were obtained from WiCell Research Institute (National Stem Cell Bank, Madison, WI, USA). The hESCs were cultured on Matrigel-coated (BD Biosciences, Stockholm, Sweden) 6-well plates (Corning, Corning, NY, USA) in mTeSR^™^1 medium (STEMCELL Technologies, Vancouver, BC, Canada), which was changed daily. Cells were maintained at 37 °C in 5% CO_2_ and passaged routinely every 3–4 days. Passaging was carried out manually by detaching the colony pieces and plating onto fresh Matrigel-coated plates. Number of passages were held between 30 and 55.

### 4.2. Embryoid Body Formation

H1 and H9 cells were used for embryoid body (EB) formation. Cells were washed gently with PBS, and TESR^™^-E6 medium (STEMCELL Technologies) was added immediately. Cells were scraped and detached manually using a pipette tip. Cell clumps were transferred onto suspension culture 6-well plates and maintained at 37 °C in 5% CO_2_. Medium was changed every other day. EBs were visualised under the microscope daily.

### 4.3. Directed Differentiation of hESCs

Directed differentiation was carried out using H9 cells without or with pre-treatment of 0.0064% DMSO (Sigma-Aldrich, Munich, Germany) or 10 nM TCDD (Cambridge Isotope Laboratories, Tewksbury, MA, USA) in mTESR^™^1 medium for 3 days. Cells were washed with PBS and harvested by GCDR (Gentle Cell Dissociation Reagent, STEMCELL Technologies) and incubated at 37 °C for 8 min in 5% CO_2_. Single cell suspensions were generated by gentle pipetting and added to 1 mL KnockOut^™^ DMEM (Dulbecco’s modified Eagle medium, Thermo Fisher Scientific, Rockford, IL, USA). Remaining cells were gathered by washing the wells with 1 mL fresh KnockOut^™^ D-MEM. Cells were then centrifuged at 300× *g* for 5 min and resuspended in medium with 10 µM ROCK (Rho-associated protein kinase, Bio-Techne, Minneapolis, MN, USA) inhibitor. Cells were counted using Countess II cell counter (Thermo Fisher Scientific) and transferred to Matrigel^™^-coated 6-well plates. For neural differentiation, STEMdiff™ neural induction medium (STEMCELL Technologies) was used. Cells were differentiated for 7 days with daily medium changes according to the manufacturer’s protocol. Pre-treated hESCs were differentiated in the presence of DMSO or 10 nM TCDD. For mesodermal and endodermal differentiation, cells were initially plated in mTESR^™^1. The next day, medium was changed to STEMdiff™ Mesodermal Induction Medium or STEMdiff™ Definite Endoderm Kit Medium (both STEMCELL Technologies), respectively. Cells were differentiated for 5 days with daily medium changes according to the manufacturer’s protocol. Pre-treated hESCs were differentiated in the presence of DMSO or 10 nM TCDD. Plating densities are shown in Figure S14.

### 4.4. RNA Isolation and Measurement of mRNA Levels

RNA isolation and mRNA measurement by RT-qPCR was performed as previously described [73]. Total RNA was extracted using Blood/Cultured Cell Total RNA Purification Kit (Favorgen, Ping-Tung, Taiwan) followed by DNase I (Thermo Fisher Scientific) treatment. Reverse transcription was performed using RevertAid First Strand cDNA Synthesis Kit (Thermo Fisher Scientific) followed by quantitative PCR on LightCycler 480 instrument (Roche, Basel, Switzerland) using Maxima SYBR Green qPCR Master Mix (Thermo Fisher Scientific) and specific primers (Table S2). Reactions were performed in triplicate. PCR cycling conditions were as follows: 95 °C for 10 min, 40 cycles of 95 °C for 10 s and 60 °C for 1 min, 45–95 °C for 7 min (melting curve). The results were analysed with LightCycler 480 software, version 1.5 (Roche). Target Cp values from triplicate measurements were averaged and normalized against TATA-binding protein (TBP) reference values. Relative target gene expression (fold change from control Cp values) was calculated as described previously [74].

### 4.5. Western Blotting

Western blotting was performed as previously described [75]. H1 or H9 hESCs were lysed with RIPA buffer (10 mM Tris-HCl (pH 7.2), 150 mM NaCl, 0.1% SDS, 1% sodium deoxycholate, 1% Triton X-100, 5 mM EDTA) containing 1X protease inhibitor cocktail (Roche, Basel, Switzerland) and resolved on 10% polyacrylamide-SDS gel. Following electrophoresis, proteins were transferred to PVDF membrane (Thermo Fisher Scientific) and blocked with TBST (20 mM Tris-HCl (pH 7.5), 150 mM NaCl, 0.1% Tween 20) containing 5% non-fat milk. The membrane was incubated overnight at 4 °C with anti-AHR (SA-210, Enzo Life Sciences, Lausen, Switzerland) and anti-actin (I-19, Santa Cruz Biotechnology, Santa Cruz, CA, USA) antibodies in TBST milk. Goat anti-rabbit secondary IgG-HRP (sc-2005) and Immobilon Western chemiluminescent HRP substrate (Millipore, Burlington, MA, USA) were used to detect signals on X-ray films (Agfa, Mortsel, Belgium). Protein bands were quantified using ImageJ software.

### 4.6. Flow Cytometry

H9 cells were pretreated with DMSO or 10 nM TCDD in mTESR^™^1 medium for 3 days. Flow cytometry was performed as previously described with minor modifications [76]. Cells were washed twice with PBS, harvested with 0.5 mM EDTA and incubated at 37 °C for 10 min. Cells were resuspended in solution A (1% BSA, 2 mM EDTA, PBS), centrifuged for 5 min at 300× *g* and resuspended again in solution A. A total of 3 × 10^5^ cells were counted using Countess II, centrifuged and resuspended in 1.6% PFA (Sigma-Aldrich) for fixation. After 10 min at RT, cells were washed with PBS, centrifuged and washed with 2 mL permeabilisation buffer (eBioscience, San Diego, CA, USA). Cells were again centrifuged for at 300× *g* 5 min and resuspended in 50 µL of permeabilisation buffer containing antibodies. Antibodies used in the experiments are found in Table S3. After 45 min of incubation in dark at RT cells were counterstained with DAPI (0.5 μg/mL). Samples were filtered and analysed with FACSAria (BD Biosciences). Data was processed using BD FACS-DiVa™ (version 6.1.3, BD Biosciences). Compensation was set with single-stained probes with CompBeads (BD Biosciences) and verified with single-stained cells. Negative population was set according to FMO and isotype controls.

### 4.7. RNA-seq

H9 cells were pretreated with DMSO (control) or 10 nM TCDD in mTESR^™^1 medium for 3 days. Alternatively, H9 cells were pre-treated with DMSO or 10 nM TCDD followed by neural, mesodermal and endodermal differentiation in the presence of DMSO or 10 nM TCDD. RNA-seq libraries were prepared using TruSeq Stranded mRNA Library Prep (Illumina, San Diego, CA, USA) according to the manufacturer’s instructions. The quality of RNA samples was analysed using Qubit fluorometer (Thermo Fisher Scientific) and TapeStation software (Agilent, Santa Clara, CA, USA). NGS sequencing was carried out using NextSeq HIGH platform with 17M reads per sample sequencing depth. Three replicates were carried out for RNA-seq experiments. Sequencing reads were aligned to GRCh38 genome version using hisat2 version 2.0.4. Samtools (version 1.16) was used for converting, sorting and indexing of BAM-files [77]. FeatureCounts of the subread-2.0.1 package was used for gene level counting of the aligned reads. R-package edgeR v3.30.3 was used for normalization using TMM method and for pairwise comparison using glmLRT function to identify differentially expressed genes. Sequencing data is archived in NCBI’s Gene Expression Omnibus (GEO) database under the accession number GSE160983.

### 4.8. ChIP-seq

ChIP-seq experiments were performed using undifferentiated H9 hESCs, neural progenitor cells (differentiated for 7 days) and definitive endoderm cells (differentiated for 5 days). Cells (approximately 12 × 10^6^) were treated with DMSO (control) or 100 nM TCDD at 37 °C for 1.5 h, and 1% formaldehyde was used for cross-linking the cells at RT for 10 min. Cross-linking was stopped with glycine at RT for 5 min. Following steps until elution were performed at 4 °C. Cells were washed twice with cold PBS and centrifuged at 1000× *g* for 5 min. For nuclei isolation, pellet was resuspended in 250 µL Nuclei Lysis Buffer 1 (50 mM Hepes pH 7.9, 140 mM NaCl, 1 mM EDTA, 0.5% NP-40, 0.25% Triton X-100, 10 mM KCl) and incubated for 10 min with gentle agitation. Following centrifugation at 1350× *g* for 5 min, the pellet was resuspended in 250 µL Nuclei Lysis Buffer 2 (10 mM Tris-HCl pH 8.0, 200 mM NaCl, 1 mM EDTA, 0.5 mM EGTA) and incubated for 10 min with gentle agitation. Nuclei were centrifuged at 1350×*g* for 5 min and pellet resuspended in 100 µL SDS Lysis buffer (1% SDS, 20 mM EDTA, 50 mM Tris-HCl pH 8.0, 1× Protease inhibitor cocktail). Lysis was performed on ice for 10 min and chromatin was sheared to appropriate length fragments using BioRuptor Plus sonicator (Diagenode, Seraing, Belgium) (10 cycles of 30 sec ON/OFF). Lysates were then diluted with 10X ChIP Dilution buffer (0.01% SDS, 1.1% Triton X-100, 1.2 mM EDTA, 16.7 mM Tris-HCl pH 8.0, 167 mM NaCl, protease inhibitor cocktail) to total volume of 1 ml and centrifuged at 10,000× *g* for 10 min. Supernatant was collected, 1% was frozen for input control and 500 µL sample was incubated overnight with 8 µg anti-AHR antibody (SA-210). Lysates were incubated for 2 h with Protein G Dynabeads (Thermo Fisher Scientific). Beads were washed for 5 min with low-salt buffer (0.1% SDS, 1% Triton X-100, 2 mM EDTA, 20 mM Tris-HCl pH 8.0, 150 mM NaCl), high-salt buffer (0.1% SDS, 1% Triton X-100, 2 mM EDTA, 20 mM Tris-HCl pH 8.0, 500 mM NaCl), LiCl buffer (0,25 M LiCl, 1% NP-40, 1% sodium deoxycholate, 1 mM EDTA, 10 mM Tris-HCl pH 8.0) and twice with TE buffer (10 mM Tris-HCl pH 8.0, 1 mM EDTA). Tube switch was performed in between last two washes to enhance specificity [78]. Chromatin-antibody complexes were eluted from Dynabeads twice with 250 µL of elution buffer (50 mM Tris-HCl, pH 8.0, 1 mM EDTA, 1% SDS) at 65 °C for 15 min on a rotator and eluates were combined. NaCl at final concentration of 300 mM was added and tubes were incubated overnight at 65 °C. Proteins were digested using 40 µg/mL Proteinase K (Thermo Fisher Scientific) at 50 °C for 2 h and RNA digested using 20 µg/mL RNase A (Thermo Fisher Scientific) at 37 °C for 30 min. DNA was purified using FavorPrep™ PCR Clean-UP Kit (Favorgen).

Immunoprecipitated and input DNA (40–60 ng) was used for library preparation with Ovation Ultralow Library System Kit V2 (Tecan Genomics, Redwood City, CA, USA) according to manufacturer’s instructions. ChIP-qPCR with selected primers was performed before sequencing to test the efficiency of ChIP and library preparation. AHR binding to *CYP1B1* enhancer in response to TCDD was evaluated and considered as positive control compared to negative control regions in *CYP1B1* and *ACTB* genes (Figures S7–S9).

NGS sequencing was performed at University of Tartu, Institute of Genomics Core Facility using NextSeq500 sequencing system (Illumina). Samples were analysed by Qubit and TapeStation for additional quality control. Sequencing was completed using single-end 75 bp reads with a depth of 2 × 10^7^ reads per sample. Raw data was demultiplexed using Local Run Manager Generate FASTQ Analysis Module (version 2.0.1). Seed sequences were aligned on GRCh37/hg19 reference genome using Bowtie2 (version 2.2.3) [79], allowing one mismatch (-N1). Samtools (version 1.16) [77] was used for converting, sorting and indexing of BAM-files. bigWig files were generated by deepTools suite [80]. Used blacklist file, containing genomic regions that give high number of reads in next-generation sequencing based studies, was made by Anshul Kundaje within EnCode Project [81]. bigWig files were visualised in UCSC genome browser using custom tracks [82]. MACS2 (version 2.1.0) [83] with default parameters was used to find AHR binding sites by either individual or combined analysis of two biological replicates of immunoprecipitated samples against input samples. For the final list of AHR bound regions all identified regions were combined, merged and overlapped with blacklisted regions using BEDtools (version 2.26.0) [84]. Peaks were associated with nearby genes within 200 kb of TSS using GREAT [85]. Overlapping regions between non-treated and TCDD-treated samples were detected and visualized by Cistrome platform [86]. DAVID Bioinformatics resources [87] was used for gene ontology analysis and HOMER [88] used for motif search. Sequencing data were archived in NCBI’s Gene Expression Omnibus (GEO) database under the accession number GSE160983.

### 4.9. ATAC-seq

H9 cells were pre-treated with DMSO (control) or 10 nM TCDD in mTESR^™^1 medium for 3 days. Cells were washed once with PBS and harvested by GCDR incubation at 37 °C for 8 min. ATAC-seq was performed as described before [89] with minor modifications. A total of 5 × 10^4^ cells were divided into separate tubes and centrifuged at 500× *g* for 5 min at 4 °C. Cells were washed once with 50 µL of cold PBS buffer and centrifuged at 500× *g* for 5 min at 4 °C. Lysis was performed by gently resuspending cell pellet in 50 µL cold lysis buffer (10 mM Tris-HCl, pH 7.4, 10 mM NaCl, 3 mM MgCl_2_, 0.1% NP-40) followed by immediate centrifugation at 500× *g* for 10 min at 4 °C. Supernatant was discarded and nuclei were resuspended in transposition reaction mix (12.5 µl 2× Tagment DNA buffer, 1.25 µL Tn5 Transposase, 11.25 µL Nuclease Free H_2_O) and incubated at 37 °C for 30 min. DNA was purified using FavorPrep™ PCR Clean-UP Kit (Favorgen) and eluted in 30 µL Elution Buffer (10 mM Tris buffer, pH 8). To amplify transposed DNA fragments, PCR reactions were performed containing 30 µL Transposed DNA, 3.5 µL Nuclease Free H2O, 2.5 µL Customized Nextera PCR universal Ad_noMX primer (25 µM), 2.5 µL Customized Nextera PCR specific index primer (25 µM), 10 µL 5× Phusion Green HF Buffer, 1 µL 10 mM dNTP, 0.5 µL Phusion DNA Polymerase (Thermo Fisher Scientific). PCR reactions were carried out as follows: 72 °C for 5 min, 98 °C for 30 s, 12 cycles of 98 °C for 10 s and 63 °C for 30 s, followed by 72 °C for 1 min. Amplified library was purified using FavorPrep™ PCR Clean-UP Kit. Qubit and TapeStation analysis was used for library quality control. Libraries were sequenced using NextSeq MID platform using paired-end 2 × 75 bp reads with a depth of 20M per sample. Further data analysis, (alignment, bam and bigwig file creation and visualization) was performed as described for ChIP-seq. Differentially open regions were identified using MACS2 bdgdiff function using default parameters. Sequencing data is archived in NCBI’s Gene Expression Omnibus (GEO) database under the accession number GSE160983.

### 4.10. Statistical Analysis

Student’s t-test was used to compare mean differences between two experimental groups. One-way ANOVA followed by Tukey’s post-hoc test was used for multiple comparisons between treatment groups. The level of statistical significance was established at *p* value of < 0.05. Data are expressed as means ± SEM.

## Figures and Tables

**Table 1:** Differentially expressed genes in hESCs (TCDD/DMSO) associated with AHR binding (“+”).

RNA-seq			ChIP-seq		
Gene	Fold Change	*p*-Value	AHR Binding		Distance Relative to TSS
			DMSO	TCDD	
*AHRR*	2.26	3.50 × 10^−3^		+	+69,749
				+	+95,863
*APOL3*	3.6	2.40 × 10^−2^		+	+15,455
*CASQ1*	1.94	3.50 × 10^−2^		+	−18,394
*CCKBR*	1.64	1.00 × 10^−2^		+	+44,133
*CTIF*	1.41	2.60 × 10e^−2^		+	−539
*CUZD1*	2.54	1.70 × 10e^−6^	+	+	−25,162
*CYP1A1*	10	8.40 × 10^−11^		+	−702
*CYP1B1*	2.73	2.00 × 10^−3^		+	−779
*EXOC2*	1.38	1.10 × 10^−3^	+	+	+9040
*GALNT5*	4.04	2.10 × 10^−3^		+	−11,039
*LHX1*	2.1	4.00 × 10^−2^	+	+	+129,491
*LHX4*	3.64	3.20 × 10^−5^		+	−168
				+	+912
*LINC00886*	4.21	4.5 × 10^−6^	+	+	0
*LRAT*	8.57	7.70 × 10^−7^		+	−2640
			+	+	−1296
				+	−212
*LRP4*	1.3	2.80 × 10^−2^		+	+14,565
*LRRTM3*	2.58	3.00 × 10^−2^		+	−32,899
*MYADML2*	5.4	3.70 × 10^−6^		+	+5254
*PCDH8*	3.02	5.50× 10^−3^		+	−3158
*PDP1*	1.66	1.90 × 10^−3^		+	−70,169
				+	−448
*PKNOX2*	1.52	3.30 × 10^−2^		+	+98,739
*PXDNL*	4.5	1.4 × 10^−4^		+	+191,524
*RORA*	1.9	2.48 × 10^−5^	+	+	−177,729
*SIX3*	2.46	6.10 × 10^−3^		+	−11,295
*SIX6*	9.43	1.50 × 10^−10^		+	+4316
*SLC16A12*	2.41	6.00 × 10^−3^		+	+1255
*SLC27A2*	1.6	2.10 × 10^−2^		+	+47
*TIPARP*	4.82	1.34 × 10^−34^	+	+	+142,217
*TESC*	1.66	3.60 × 10^−3^		+	+46,627

**Table 2:** Differentially expressed genes in definitive endoderm cells (TCDD/DMSO) associated with AHR binding (“+”).

RNA-seq			ChIP-seq		
Gene	Fold Change	*p*-Value	AHR Binding (hESC)		Distance Relative to TSS
			DMSO	TCDD	
*CDCA7L*	1.39	2.9 × 10^−2^	+	+	−120,620
*CUZD1*	−10.16	4.2 × 10^−2^	+	+	−25,162
*MYADML2*	−28.61	7.4 × 10^−4^		+	+5254
*S1PR1*	2.55	3.0 × 10^−2^		+	+73,148

**Table 3:** Differentially expressed genes in early mesoderm cells (TCDD/DMSO) associated with AHR binding (“+”).

RNA-seq			ChIP-seq						
Gene	Fold Change	*p*-Value	AHR Binding						Distance Relative to TSS
			hESC		Endo		Neur		
			DMSO	TCDD	DMSO	TCDD	DMSO	TCDD	
*ADM*	−2.59	4.9 × 10^−2^		+					+46,975
*AK4*	1.75	2.9 × 10^−2^		+					+131,446
*AOAH*	−3.9	3.2 × 10^−2^						+	+65,017
*CBX4*	−4.6	2.8 × 10^−5^		+					−110,655
*CCNG1*	1.32	3.6 × 10^−2^		+					+387
*CRABP1*	3.1	3.8 × 10^−2^						+	+3175
*CYP1A1*	−9.07	4.1 × 10^−2^		+				+	−702
*EPC2*	−1.43	1.3 × 10^−2^	+	+	+	+		+	+450
*FGFR3*	1.71	1.8 × 10^−2^		+					−15,013
*GADD45G*	−4.29	2.0 × 10^−3^		+					+72,997
*GSE1*	−1.70	6.9 × 10^−3^		+				+	−29,402
*HSPB8*	−5.34	1.5 × 10^−3^							+124,278
*LRAT*	3.79	1.6 × 10^−3^		+					−2640
			+	+				+	−1296
				+					−212
*MAP3K8*	1.51	2.2 × 10^−2^		+				+	+2436
*SLC7A5*	−1.91	4.4 × 10^−2^		+					−1151
*SLC25A25*	−1.46	3.2 × 10^−2^	+		+			+	−896

**Table 4:** Differentially expressed genes in neural progenitors (TCDD/DMSO) associated with AHR binding (“+”).

RNA-seq			ChIP-seq						
Gene	Fold Change	*p*-Value	AHR Binding						Distance Relative to TSS
			hESC		Endo		Neur		
			DMSO	TCDD	DMSO	TCDD	DMSO	TCDD	
*AHRR*	3.95	2.2 × 10^−6^		+				+	+69,749
				+				+	+95,863
*C3orf80*	−9.2	4.7 × 10^−3^		+					−186,661
*CCDC60*	1.84	2.7 × 10^−2^						+	−31,792
*CCKBR*	2.45	2.3 × 10^−6^		+				+	+44,133
*CRYBB1*	−2.84	4.8 × 10^−2^	+	+				+	−30,267
*CYP1A1*	34.78	1.4 × 10^−30^		+				+	−702
*CYP1B1*	7.6	1.2 × 10^−9^						+	−20,263
				+				+	−779
*CYP27A1*	1.65	2.2 × 10^−2^						+	+24,031
*GRB7*	2.84	2.0 × 10^−5^						+	+62,663
*HS3ST5*	3.07	2.6 × 10^−6^	+	+					−20,093
*IKZF3*	7.62	1.0 × 10^−3^						+	+62,753
*LINC00886*	2.02	7.5 × 10^−3^	+	+	+	+		+	0
*LRAT*	3.85	1.3 × 10^−3^		+					−2640
			+	+				+	−1296
				+					−212
*PDP1*	1.49	1.5 × 10^−2^		+				+	−70,169
				+					−448
*TPRA1*	1.44	2.4 × 10^−3^	+	+	+	+		+	−376
*RUNX1T1*	3.38	1.3 × 10^−2^			+	+		+	−8068
*TIPARP*	2.27	6.96 × 10^−9^	+	+	+	+		+	+142,217
*TSC22D1*	1.34	4.0 × 10^−2^						+	+179,610

## Supplementary Materials

Supplementary Materials can be found at https://www.mdpi.com/1422-0067/21/23/9052/s1.

## Abbreviations

AHR	Aryl hydrocarbon receptor
AIP	Aryl hydrocarbon receptor interacting protein
ARNT	Aryl hydrocarbon receptor nuclear translocator
bHLH/PAS	Basic helix-loop-helix/PER-ARNT-SIM
DMSO	Dimethyl sulfoxide
EB	Embryoid body
EXOC2	Exocyst complex component 2
hESC	Human embryonic stem cell
HSP90	Heat shock protein 90
LRAT	Lecithin retinol acyltransferase
MAP2	Microtubule-associated protein 2
RORA	Retinoic acid receptor-related orphan receptor alpha
TCDD	2,3,7,8-tetrachlorodibenzo-p-dioxin
TH	Tyrosine hydroxylase
TIPARP	TCDD-inducible poly(ADP-ribose) polymerase
