# Supplementary material for: Impact of AHR Ligand TCDD on Human Embryonic Stem Cells and Early Differentiation
Source: Int J Mol Sci. 2020 Nov 28;21(23):9052. doi: 10.3390/ijms21239052 (PMC7731104; doi:10.3390/ijms21239052)
Supplement: Supplementary file 1 [file ijms-21-09052-s001.zip › Supplementary Figures.pdf]

Supplementary figures S1-S14 and supplementary tables S1-S3

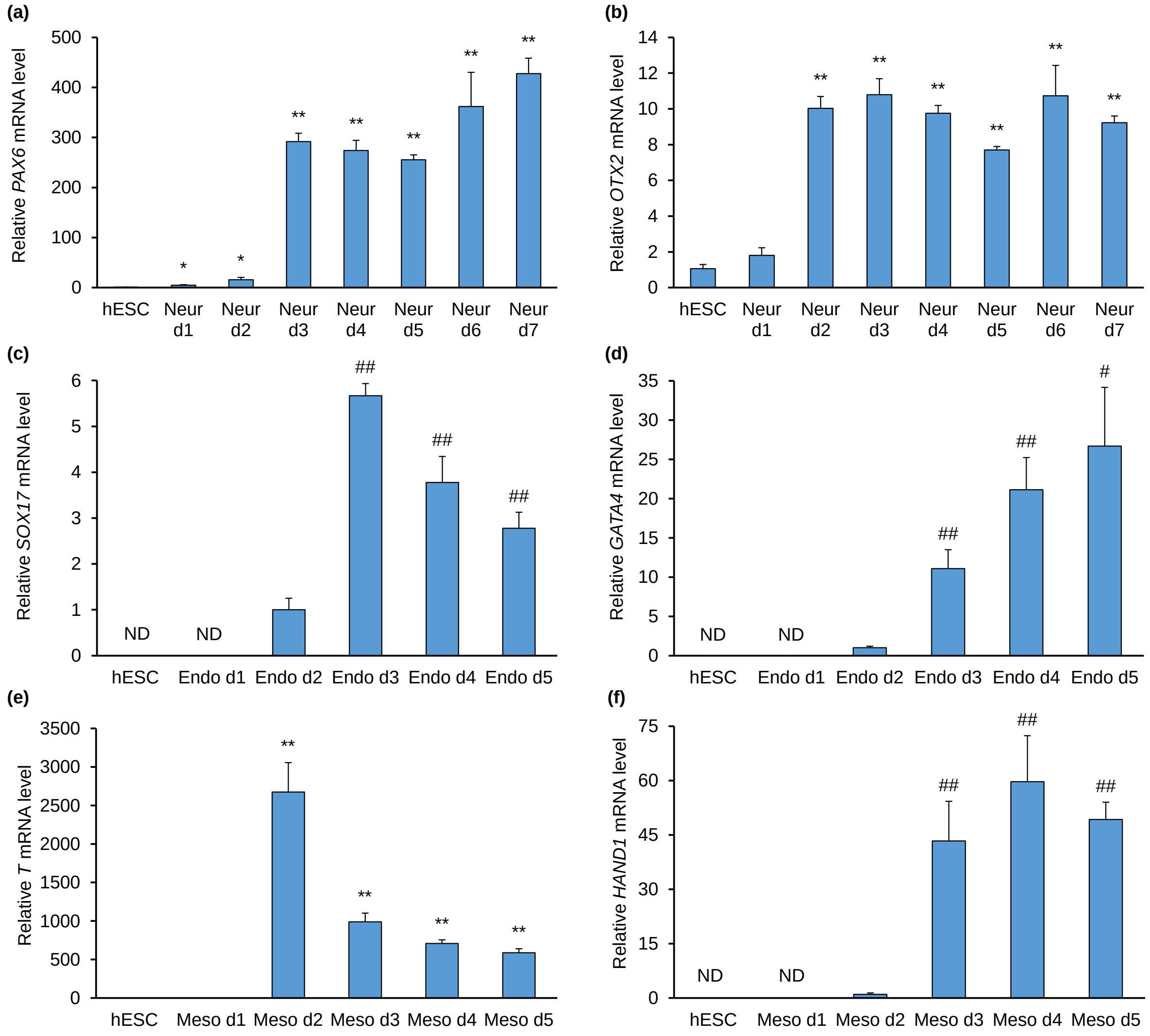

Figure S1. Analysis of differentiation marker gene expression in H9 hESCs and during directed differentiation into neural, endo- and mesodermal lineages. qPCR analysis of neural marker genes *PAX6* (**a**) and *OTX2* (**b**), endodermal marker genes *SOX17* (**c**) and *GATA4 (**d**)* and mesodermal marker genes *T (**e**)* and *HAND1 (**f**)* mRNA levels. Data are presented relative to hESC (**a**, **b**, **e**) or d2 (**c**, **d**, **f**) as means ± SEM from three independent experiments. *$p < 0.05$ vs hESC; **$p < 0.01$ vs hESC; #$p < 0.05$ vs d2; ##$p < 0.01$ vs d2.

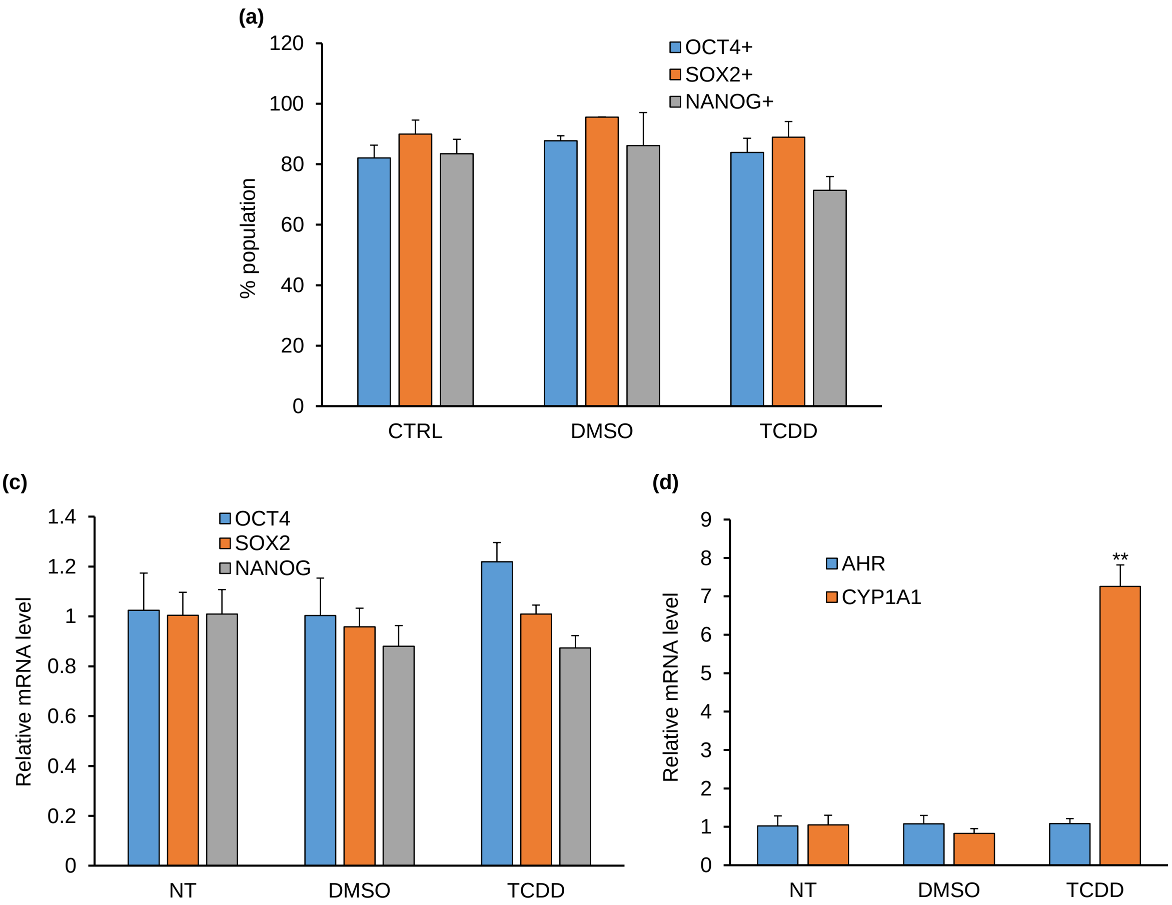

Figure S2. Impact of TCDD on pluripotency. hESCs were treated with 10 nM TCDD for 3 days. (**a**) Flow cytometry analysis of OCT+, SOX+ and NANOG+ cells. qPCR analysis of *OCT4*, *SOX2*, *NANOG* (**b**), *AHR* and *CYP1A1* (**c**) mRNA levels. Data are presented relative to NT (non-treated) as means ± SEM from three independent experiments. $^{**}p < 0.01$.

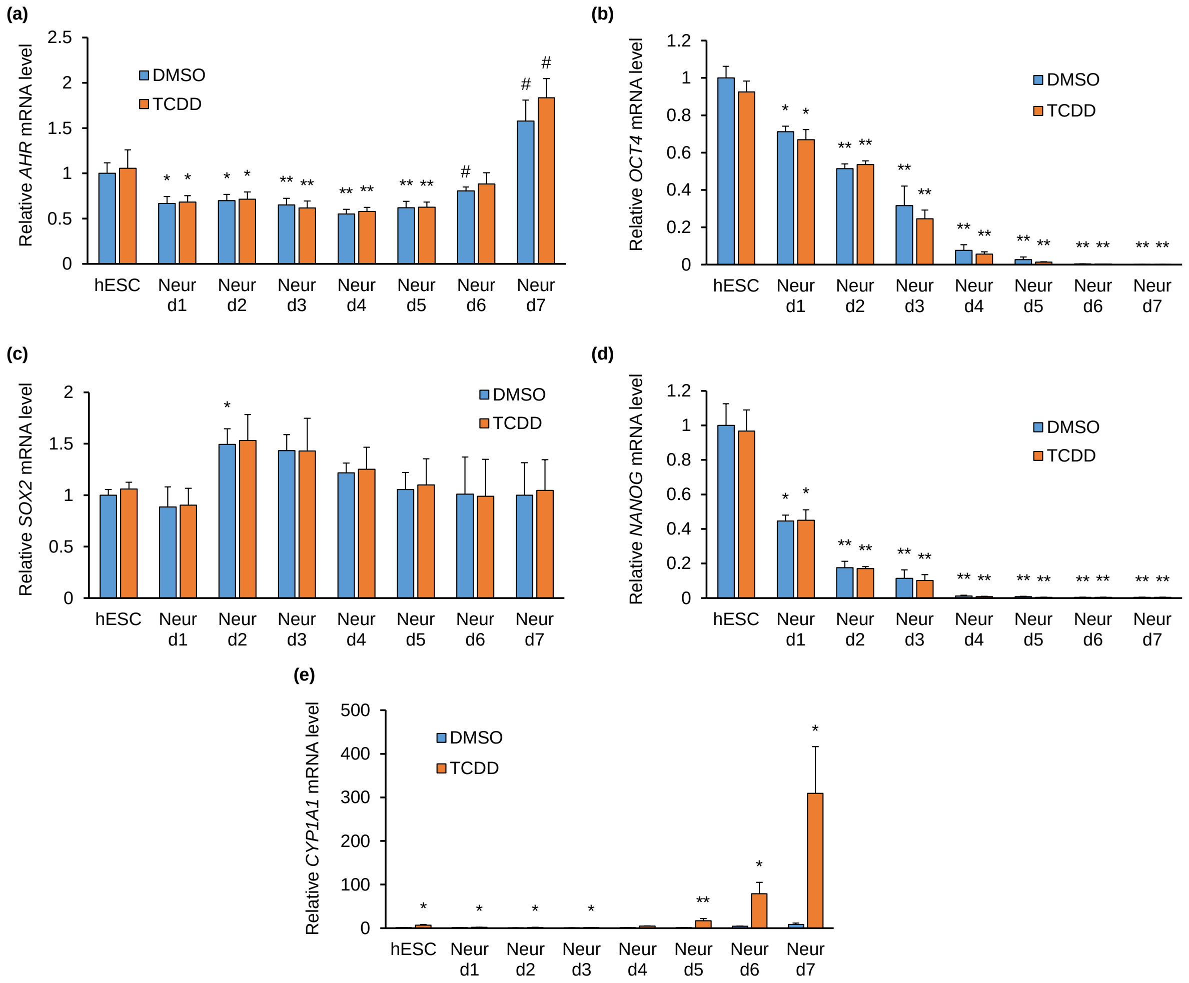

Figure S3. Analysis of *AHR, CYP1A1* and pluripotency gene expression in DMSO and 10 nM TCDD pre-treated H9 hESCs and during differentiation into neural lineage. qPCR analysis of *AHR* (**a**), *OCT4* (**b**), *SOX2* (**c**), *NANOG* (**d**) *and CYP1A1* (**e**) mRNA levels. Data are presented relative to hESC DMSO as means ± SEM from three independent experiments. *$p < 0.05$; **$p < 0.01$.

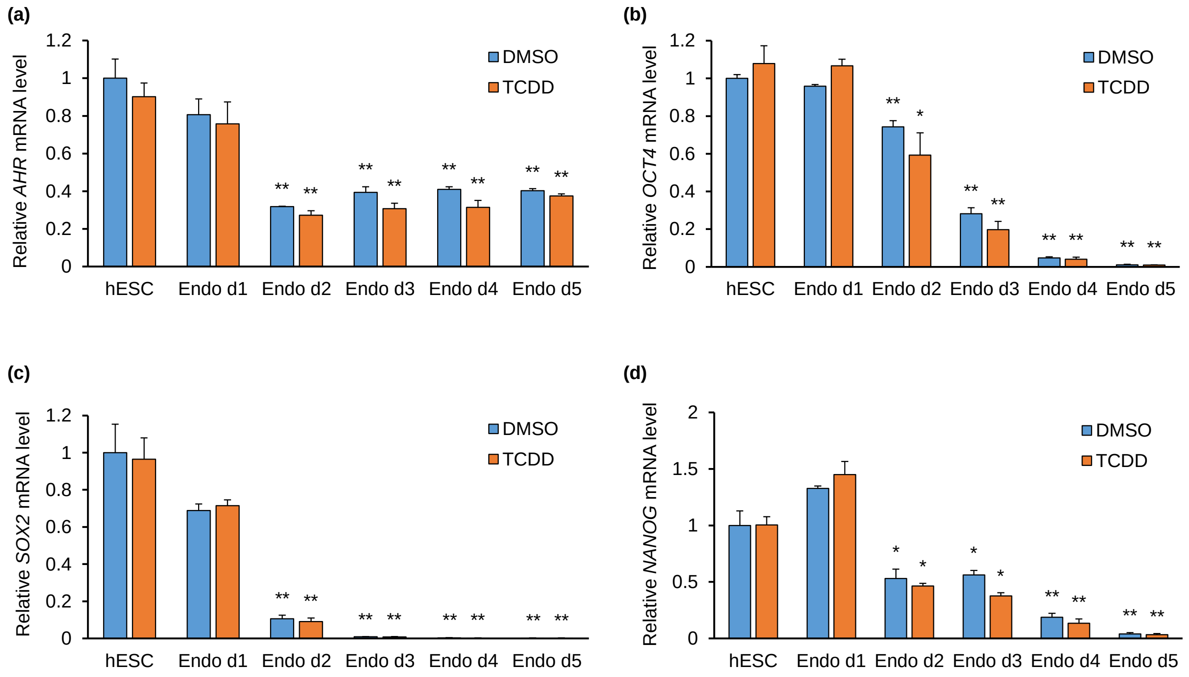

Figure S4. Analysis of *AHR* and pluripotency gene expression in DMSO and 10 nM TCDD pre-treated H9 hESCs and during differentiation into endodermal lineage. qPCR analysis of *AHR* (**a**), *OCT4* (**b**), *SOX2* (**c**) and *NANOG* (**d**) mRNA levels. Data are presented relative to hESC DMSO as means ± SEM from three independent experiments. *$p < 0.05$, **$p < 0.01$.

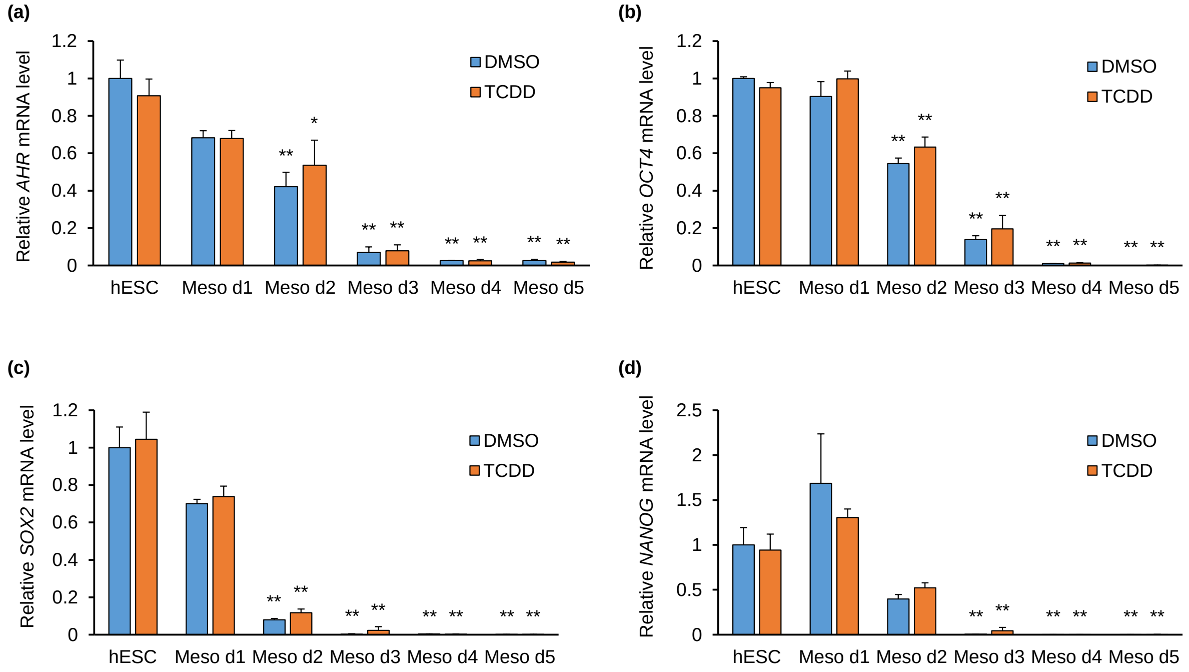

Figure S5. Analysis of *AHR* and pluripotency gene expression in DMSO and 10 nM TCDD pre-treated H9 hESCs and during differentiation into mesodermal lineage. qPCR analysis of *AHR* (**a**), *OCT4* (**b**), *SOX2* (**c**) and *NANOG* (**d**) mRNA levels. Data are presented relative to hESC DMSO as means ± SEM from three independent experiments. *$p < 0.05$, **$p < 0.01$.

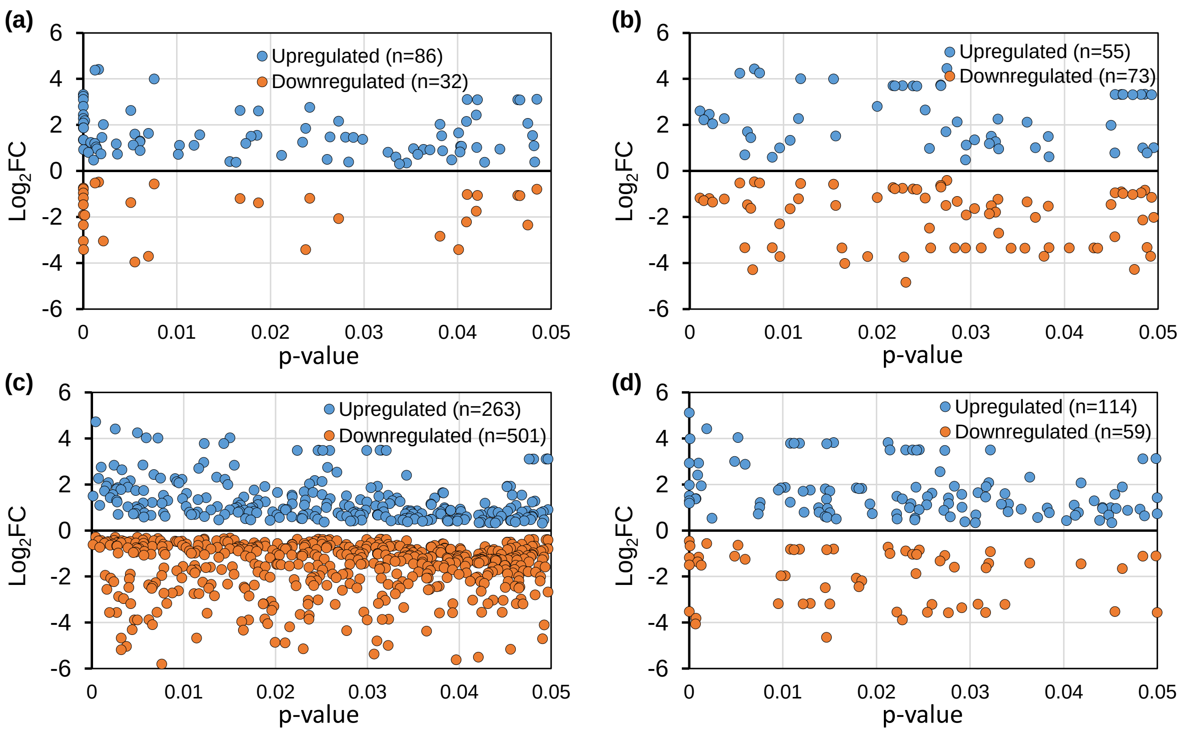

Figure S6. Impact of TCDD on global gene expression in hESCs and differentiated cells. TCDD treatment (10nM, vs DMSO) was performed on hESCs (**a**; 3-day pre-treatment), endodermal (**b**; pre-treatment + 5-day differentiation/treatment), mesodermal (**c**; pre-treatment + 5-day differentiation/treatment) and neural cells (**d**; pre-treatment + 7-day differentiation/treatment) followed by RNA-seq. Genes are plotted by p-value (X-axis) respective to the relative expression (TCDD vs DMSO, Log_2 fold change, Y-axis).

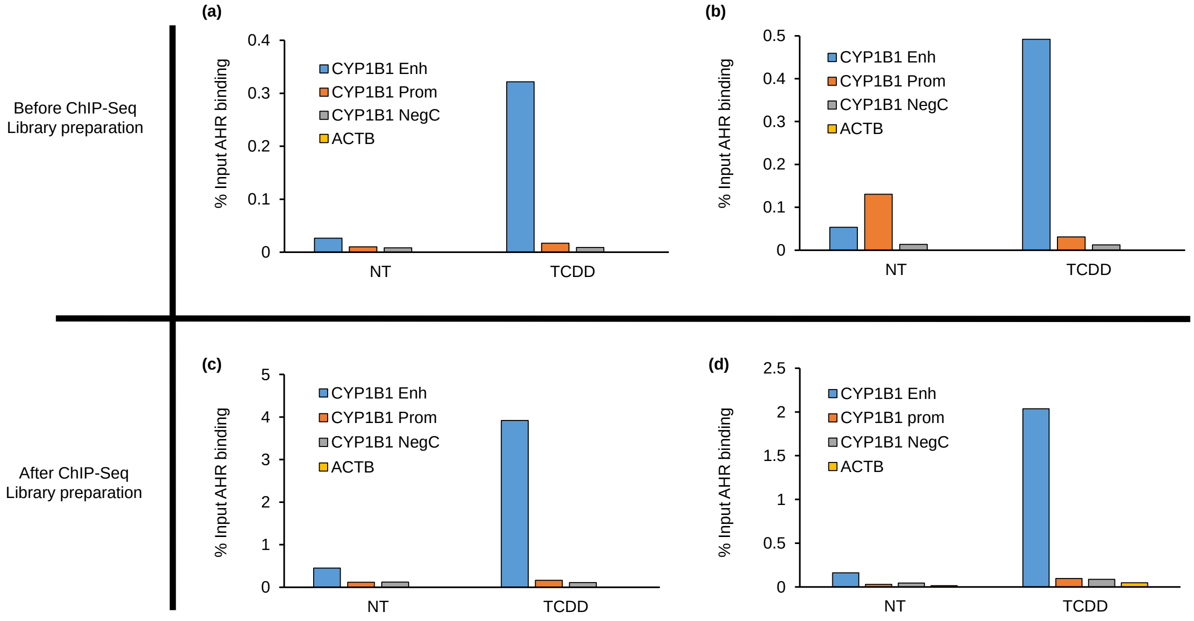

Figure S7. The effect of TCDD on AHR binding to *CYP1B1* enhancer and negative control regions in undifferentiated hESCs. ChIP-qPCR data of two independent experiments showing relative AHR binding before (**a**, **b**) and after (**c**, **d**) ChIP-Seq library preparation.

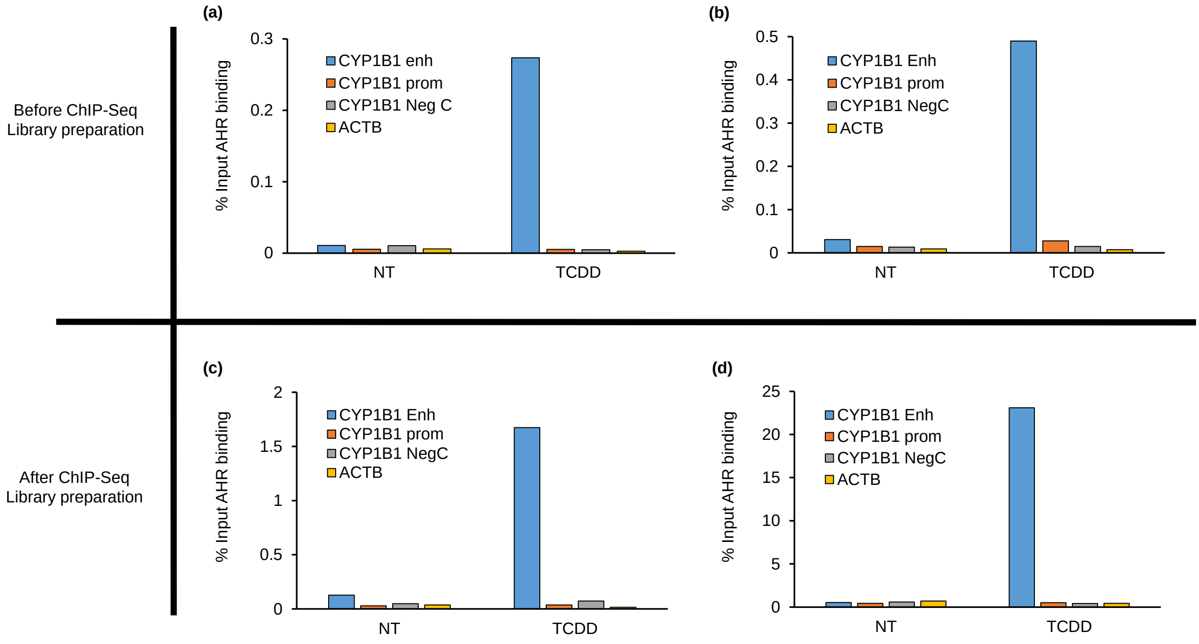

Figure S8. The effect of TCDD on AHR binding to *CYP1B1* enhancer and negative control regions in neural progenitor cells. ChIP-qPCR data of two independent experiments showing relative AHR binding before (**a**, **b**) and after (**c**, **d**) ChIP-Seq library preparation.

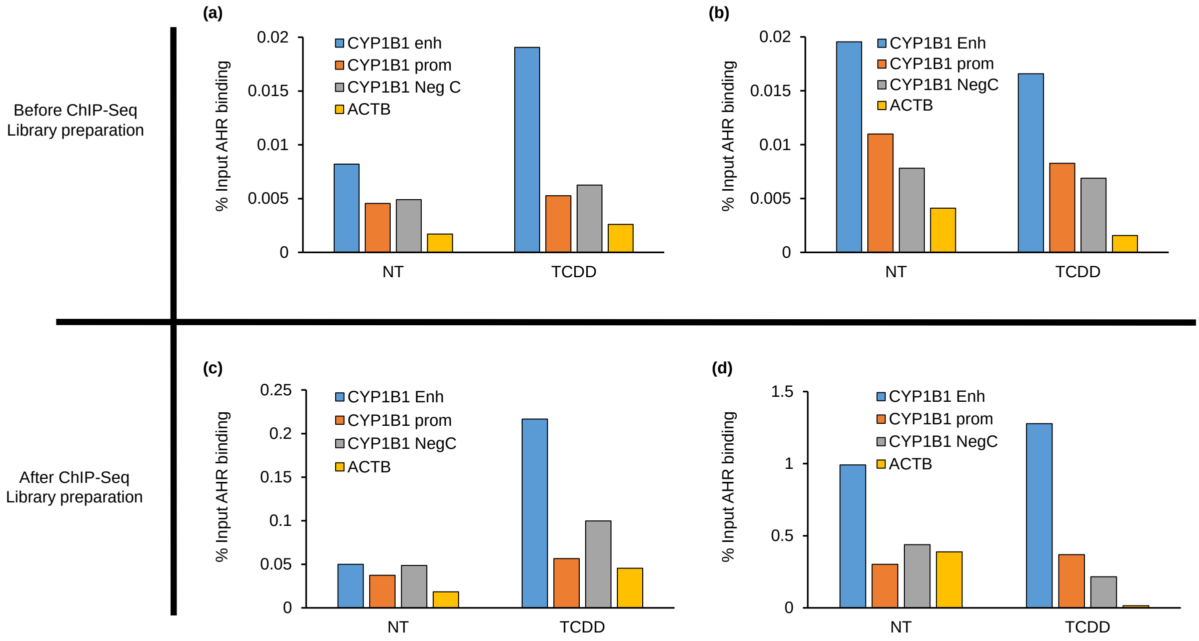

Figure S9. The effect of TCDD on AHR binding to *CYP1B1* enhancer and negative control regions in definitive endoderm cells. ChIP-qPCR data of two independent experiments showing relative AHR binding before (**a**, **b**) and after (**c**, **d**) ChIP-Seq library preparation.

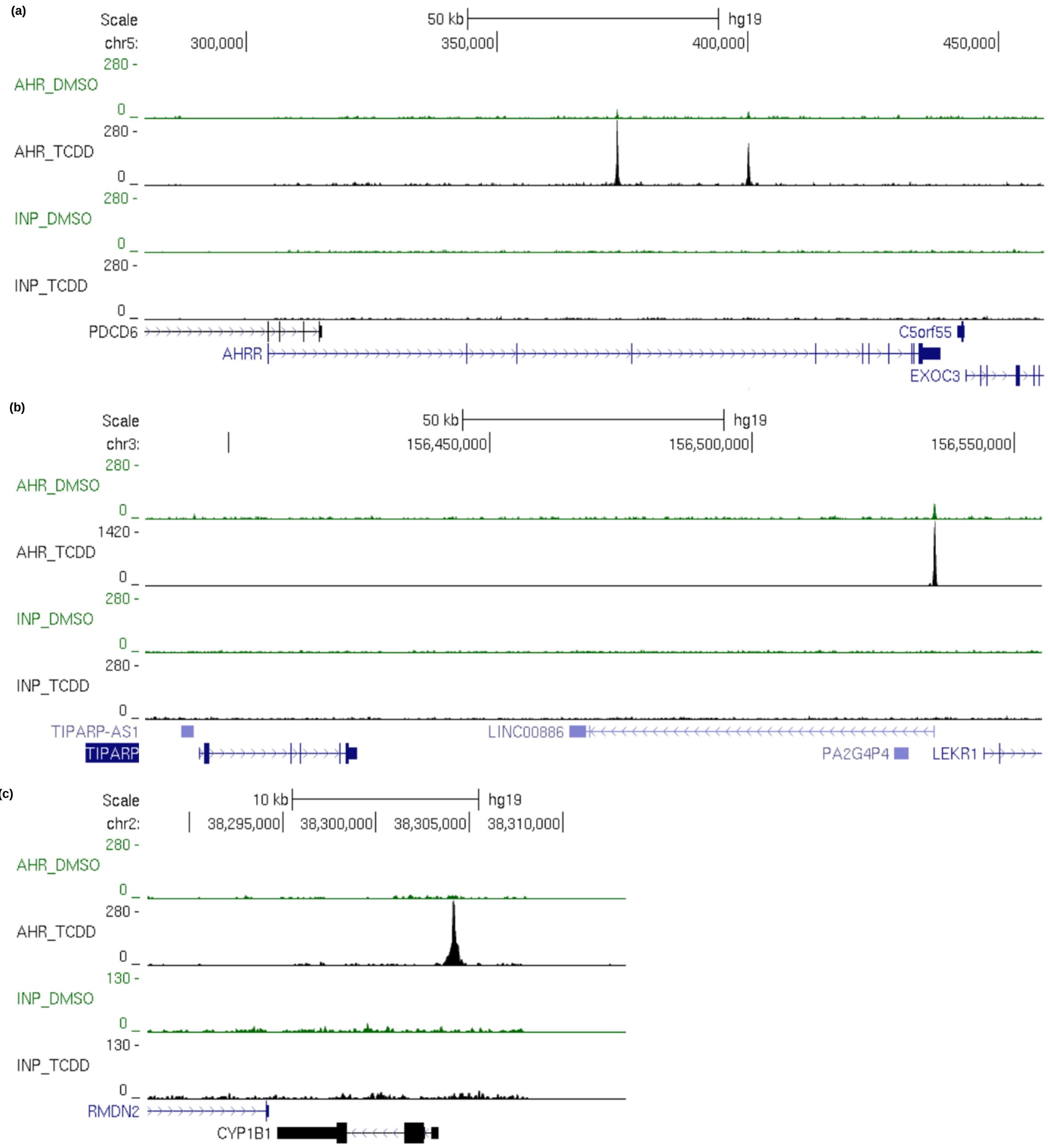

Figure S10. ChIP-seq AHR binding profile near AHR known target genes *AHRR* (**a**), *TIPARP* (**b**) and *CYP1B1* (**c**). Data is presented as fragment pileup and visualised in UCSC Genome browser.

Rank	Motif	P-value	log P-pvalue	% of Targets	% of Background	STD(Bg STD)	Best Match/Details
1	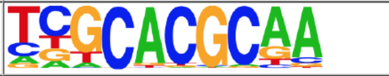	1e-157	-3.632e+02	57.02%	5.34%	36.2bp (75.2bp)	Arnt:Ahr(bHLH)/MCF7-Arnt-ChIP-Seq(Lo_et_al.)/Homer(0.959)
2	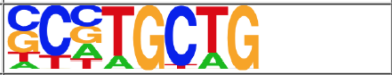	1e-21	-4.927e+01	46.63%	23.24%	51.3bp (66.9bp)	Zic1::Zic2/MA1628.1/Jaspar(0.901)
3	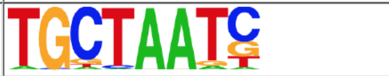	1e-18	-4.162e+01	16.01%	4.01%	54.4bp (67.9bp)	Brn1(POU,Homeobox)/NPC-Brn1-ChIP-Seq(GSE35496)/Homer(0.822)
4	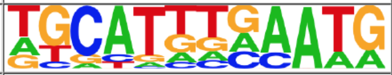	1e-17	-3.929e+01	15.45%	3.94%	52.2bp (67.8bp)	Pou5f1::Sox2/MA0142.1/Jaspar(0.775)
5	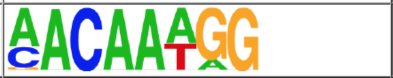	1e-15	-3.488e+01	25.00%	10.11%	54.7bp (64.0bp)	Sox3(HMG)/NPC-Sox3-ChIP-Seq(GSE33059)/Homer(0.964)
6	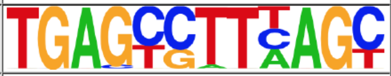	1e-15	-3.474e+01	3.09%	0.06%	42.4bp (55.2bp)	PB0049.1_Nr2f2_1/Jaspar(0.607)
7	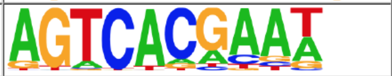	1e-14	-3.244e+01	8.15%	1.28%	49.3bp (61.6bp)	Npas4(bHLH)/Neuron-Npas4-ChIP-Seq(GSE127793)/Homer(0.796)
8	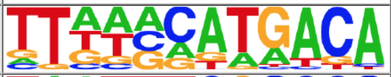	1e-13	-3.217e+01	13.48%	3.64%	61.6bp (65.6bp)	MEIS1/MA0498.2/Jaspar(0.721)
9	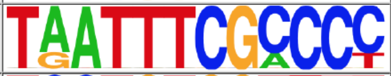	1e-12	-2.916e+01	1.40%	0.00%	39.6bp (0.0bp)	PH0037.1_Hdx/Jaspar(0.715)
10	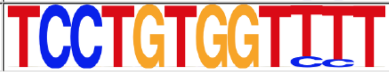	1e-12	-2.815e+01	1.97%	0.02%	42.2bp (49.3bp)	RUNX1/MA0002.2/Jaspar(0.807)

Figure S11. DNA motif analysis by HOMER motif discovery software of combined enriched regions in ChIP-seq of 3 cell-types.

Table S1. Overlapping ChIP-seq peaks in DMSO and/or TCDD treated cells (associated genes).

Ensemble	Gene	hESC	Endo	Neur
ENSG00000122545	SEPTIN7	+	+	+
ENSG00000059573	ALDH18A1	+	+	+
ENSG00000002330	BAD	+	+	+
ENSG00000157388	CACNA1D	+	+	+
ENSG00000122565	CBX3	+	+	+
ENSG00000134780	DAGLA	+	+	+
ENSG00000100201	DDX17	+	+	+
ENSG00000100697	DICER1	+	+	+
ENSG00000135999	EPC2	+	+	+
ENSG00000122566	HNRNPA2B1	+	+	+
ENSG00000171169	NAIF1	+	+	+
ENSG00000272325	NUDT3	+	+	+
ENSG00000117461	PIK3R3	+	+	+
ENSG00000148339	SLC25A25	+	+	+
ENSG00000183048	SLC39A10	+	+	+
ENSG00000165209	STRBP	+	+	+
ENSG00000163870	TPRA1	+	+	+
ENSG00000163659	TIPARP	+	+	+
ENSG00000240875	LINC00886	+	+	+
ENSG00000152213	ARL11	+	+	
ENSG00000103326	CAPN15	+	+	

ENSG00000123179	EBPL	+	+	
ENSG00000110321	EIF4G2	+	+	
ENSG00000170296	GABARAP	+	+	
ENSG00000095015	MAP3K1	+	+	
ENSG00000052802	MSMO1	+	+	
ENSG00000040633	PHF23	+	+	
ENSG00000142784	WDTC1	+	+	
ENSG00000130227	XPO7	+	+	
ENSG00000168010	ATG16L2		+	+
ENSG00000170558	CDH2		+	+
ENSG00000138668	HNRNPD		+	+
ENSG00000079102	RUNX1T1		+	+
ENSG00000063438	AHRR	+		+
ENSG00000221990	C5orf55	+		+
ENSG00000110148	CCKBR	+		+
ENSG00000164649	CDCA7L	+		+
ENSG00000150394	CDH8	+		+
ENSG00000196431	CRYBA4	+		+
ENSG00000100122	CRYBB1	+		+
ENSG00000140465	CYP1A1	+		+
ENSG00000138061	CYP1B1	+		+
ENSG00000112685	EXOC2	+		+
ENSG00000131149	GSE1	+		+
ENSG00000164818	HEATR2	+		+
ENSG00000188996	HUS1B	+		+
ENSG00000134504	KCTD1	+		+
ENSG00000121454	LHX4	+		+
ENSG00000121207	LRAT	+		+
ENSG00000151033	LYZL2	+		+
ENSG00000107968	MAP3K8	+		+
ENSG00000136099	PCDH8	+		+
ENSG00000164951	PDP1	+		+
ENSG00000170955	PRKCDBP	+		+
ENSG00000170955	CAVIN3	+		+
ENSG00000138083	SIX3	+		+
ENSG00000153498	SPACA7	+		+
ENSG00000164828	SUN1	+		+
ENSG00000164953	TMEM67	+		+

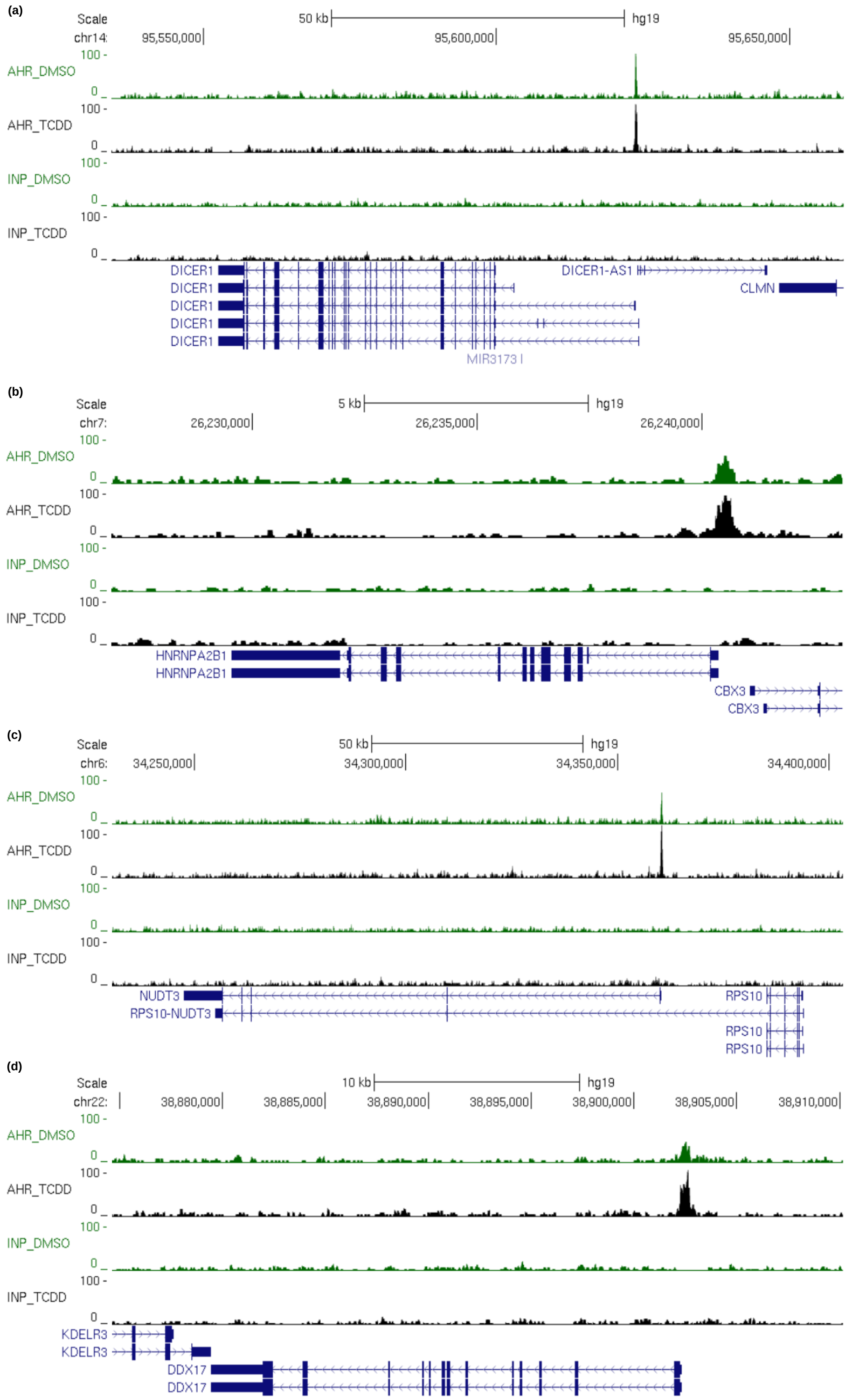

Figure S12. ChIP-seq AHR binding profile in hESCs near *DICER1* (**a**), *HNRNPA2B1* (**b**), *NUDT3* (**c**) and *DDX17* (**d**). Data is presented as fragment pileup and visualised in UCSC Genome browser.

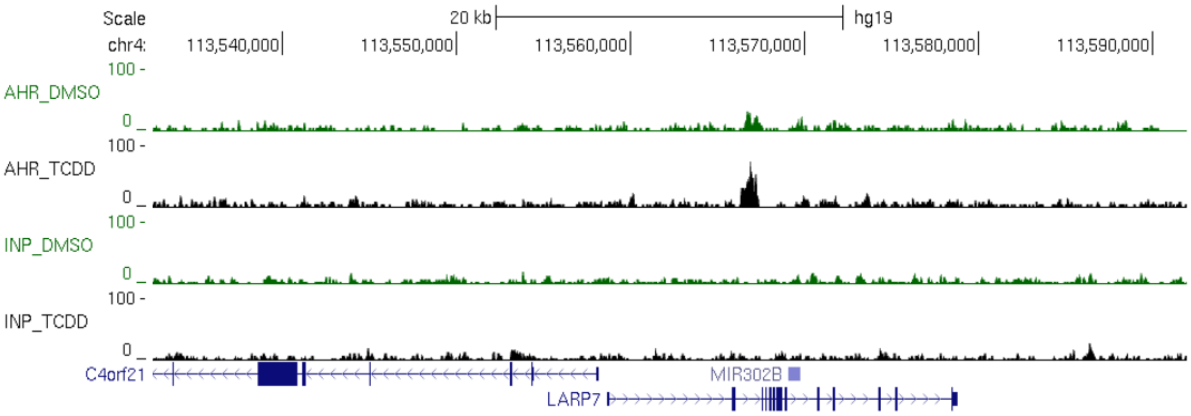

Figure S13. ChIP-seq AHR binding profile in hESCs near miR-302B. Data is presented as fragment pileup and visualised in UCSC Genome browser.

(**a**)

**Day**	**d1**	**d2**	**d3**	**d4**	**d5**	**d6**	**d7**
**Number of cells**	$2x10^6$	$2x10^6$	$1x10^6$	$1x10^6$	$7.5x10^5$	$5x10^5$	$5x10^5$

(**b**)

**Day**	**d1**	**d2**	**d3**	**d4**	**d5**
**Number of cells**	$2x10^6$	$1.5x10^6$	$7.5x10^5$	$3x10^5$	$2.5x10^5$

(**c**)

**Day**	**d1**	**d2**	**d3**	**d4**	**d5**
**Number of cells**	$2x10^6$	$2x10^6$	$2x10^6$	$2x10^6$	$2x10^6$

Figure S14. Plating densities of cells for differentiation. Neural **(a)**, mesodermal **(b)** and endodermal **(c)** lineage. Indicated are numbers of cells plated on day 0 for analysis on the specified day.

Table S2. Oligonucleotides used in the study.

**Application**	**Oligonucleotide**		**Sequence (5′–3′)**
	AHR mRNA	Forward	ATTACAGGCTCTGAATGGCTTTG
		Reverse	TGACATCAGACTGCTGAAACCCTAG
	OCT4 mRNA	Forward	CTGGAGCAAAACCCGGAGG
		Reverse	CCTCAAAGCGGCAGATGGTC
	SOX2 mRNA	Forward	CATGCACCGCTACGACG
		Reverse	CGGACTTGACCACCGAAC
	NANOG mRNA	Forward	CCTGTGATTTGTGGGCCTG
		Reverse	GACAGTCTCCGTGTGAGGCAT
	PAX6 mRNA	Forward	AACAGACACAGCCCTCACAAAC
		Reverse	CGGGAACTTGAACTGGAACTGAC

RT-qPCR	*OTX2* mRNA	Forward	GCTGGCTATTTGGAATTTAAAGC
		Reverse	GGGTTTGGAGCAGTGGAAC
	T mRNA	Forward	ATGACAATTGGTCCAGCCTT
		Reverse	CGTTGCTCACAGACCACAG
	HAND1 mRNA	Forward	GTGAGAGCAAGCGGAAAAG
		Reverse	GTGCGTCCTTTAATCCTCTTC
	GSC mRNA	Forward	GAGGGAAGAGGAAGGTAAAAG
		Reverse	AAGTAATACGGGCAAGTGTCC
	SOX17 mRNA	Forward	GGCGCAGCAGAATCCAGA
		Reverse	CCACGACTTGCCCAGCAT
	GATA4 mRNA	Forward	GCTCCTTCAGGCAGTGAGAG
		Reverse	CTGTGCCCGTAGTGAGATGA
	CYP1A1 mRNA	Forward	GCTGACTTCATCCCTATTCTTCG
		Reverse	TTTTGTAGTGCTCCTTGACCATCT
	TBP mRNA	Forward	TGCACAGGAGCCAAGAGTGAA
		Reverse	CACATCACAGCTCCCCACCA
ChIP-qPCR	*CYP1B1* promoter	Forward	TTTTGAGGCTGGAAAACAGGTACT
		Reverse	ACGTGATGACGCAGGACGTA
	CYP1B1 enhancer	Forward	GCGGCAAACCTCTGAACTTC
		Reverse	TGATCCCCAGGCTGAGTCAT
	CYP1B1 NegC	Forward	GCCTCCTTCTCCTGATTTGGA
		Reverse	CTGCCATGGGAAATGAGGG
	ACTB	Forward	GCCTCCTTCTCCTGATTTGGA
		Reverse	CTGCCATGGGAAATGAGGG

Table S3. Antibodies used in flow cytometry.

Target	Dilution	Source/Isotype	Manufacturer
OCT4 (Alexa 647)	1:100	Mouse monoclonal IgG2b	BioLegend
NANOG (PE)	1:5	Mouse monoclonal IgG1	BD Biosciences
SOX2 (PerCP-Cy5.5)	1:20	Mouse monoclonal IgG1	BD Biosciences
**Isotype controls**			
Alexa 647	1:100	Mouse IgG2b	BioLegend
PE	1:80	Mouse IgG1	BD Biosciences
PerCP-Cy5.5	1:5	Mouse IgG1	BD Biosciences
